# Supplementary figures and images for: Complementary encoding of spatial information in hippocampal astrocytes
Source: PLoS Biol. 2022 Mar 3;20(3):e3001530. doi: 10.1371/journal.pbio.3001530 (PMC8893713; doi:10.1371/journal.pbio.3001530)

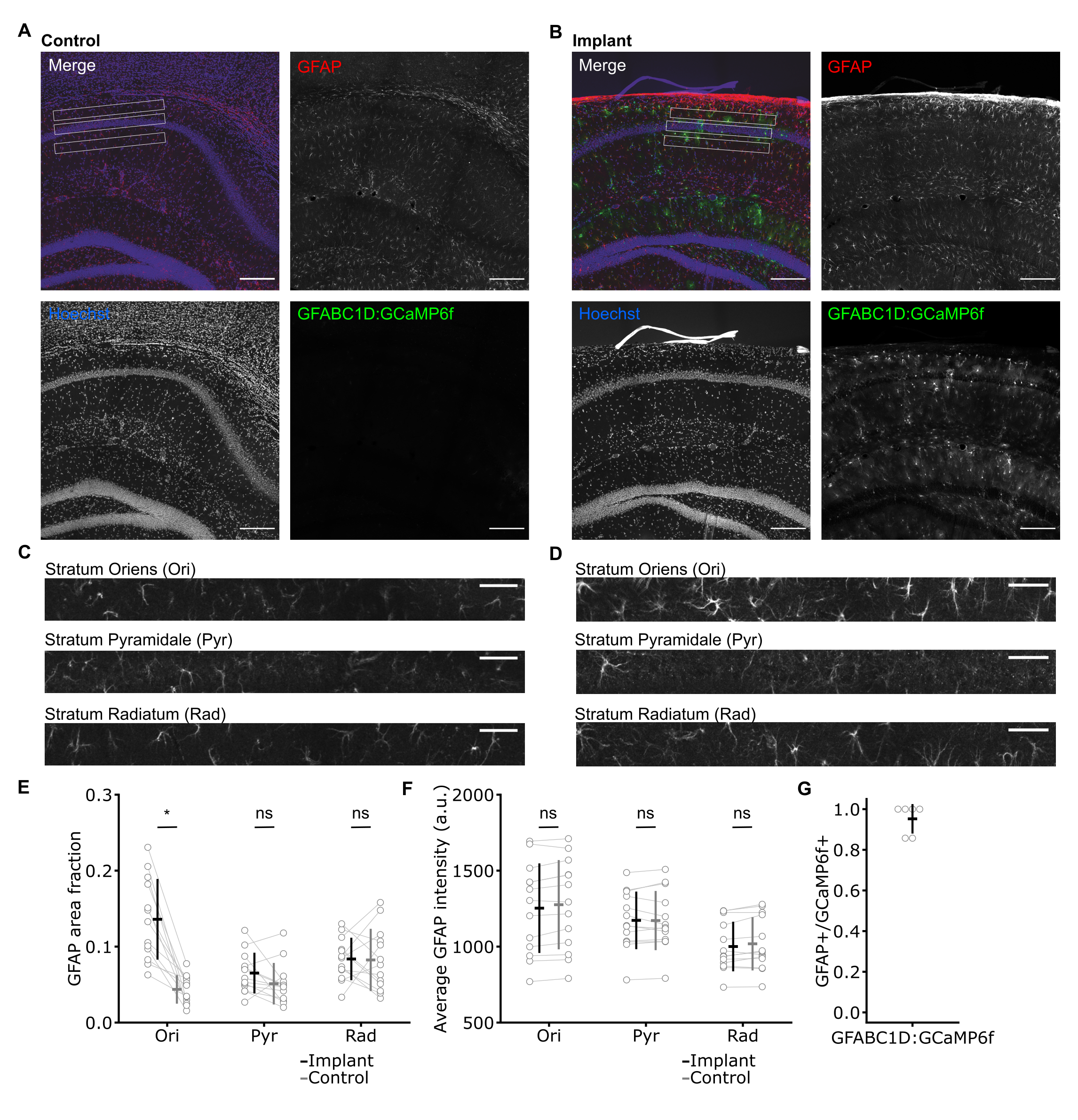

Supplement: S1 Fig — (A, B) Representative images of hippocampal brain slices from animals injected with AAV5 pZac2.1 gfaABC1D-cyto-GCaMP6f and implanted with a chronic optical window. Images are maximum intensity projection of confocal z-stacks (8 planes, 1.5 μm/step) from hemispheres contralateral (A) and ipsilateral (B) to the injection and implant site. Brain slices were stained with anti-GFAP and anti-GFP primary antibodies, which were counterstained with Alexa-546 and Alexa-488 conjugated secondary antibodies, respectively. Cell nuclei were labeled with Hoechst. (C, D) Zoom-in of the ROIs (white rectangles in A and B) used for quantification of GFAP-staining in stratum Oriens, stratum Pyramidale, and stratum Radiatum. (E) Fraction of ROI area immunolabeled for GFAP. (F) Average fluorescence intensity of GFAP-positive pixels in the 3 hippocampal regions under the different experimental conditions. Data are presented as mean ± SD from 13 slices in 3 animals. In E: p = 1.4E-2, p = 1.3E-1, and p = 8.2E-1 for stratum Oriens, Pyramidale, and Radiatum, respectively. Paired t test. In F: p = 8.8E-2, p = 9.5E-1, and p = 2.0E-1 for stratum Oriens, Pyramidale, and Radiatum, respectively. Paired t test. (G) Fraction of GCaMP6f cells immunolabeled for GFAP (95 ± 7%, out of a total of 45 GCaMP6f-expressing cells from N = 6 sections from 3 mice). Scale bars: 200 μm and 50 μm for A and B and C and D, respectively. The data presented in this figure can be found in S2 Data. GFAP, glial fibrillary acidic protein; GFP, green fluorescent protein; ROI, region of interest. (TIFF) [file pbio.3001530.s001.tiff]

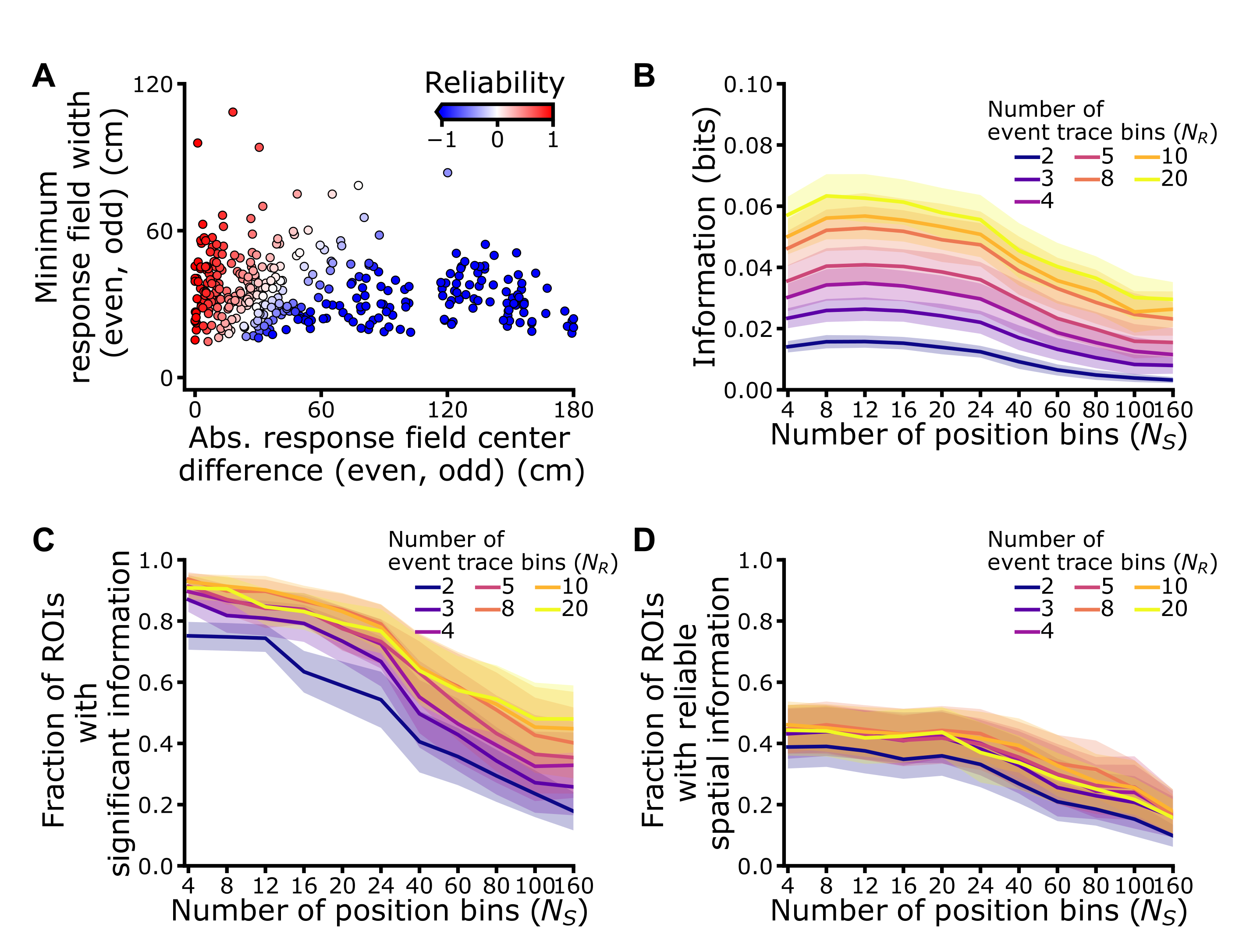

Supplement: S2 Fig — (A) Minimum response field width between even and odd trials as a function of the difference in place field position. The pseudocolor scale indicates reliability of the response (see Methods). (B, C) Mutual information values (B) and fraction of ROIs showing significant spatial information (C) as a function of the number of bins for the stimulus (animals’ position in the linear track). Colors indicate different binning of the response (calcium event trace). Mutual information values were bias-corrected using bootstrap method (104 iterations). Significance level for information content was set at p < 0.05. (D) Fraction of ROIs with reliable spatial information as a function of the number of bins for the stimulus. Colors indicate different binning of the response. Data in (B, D) are presented as mean ± SEM from 7 imaging sessions in 3 animals. The data presented in this figure can be found in S2 Data. ROI, region of interest; SEM, standard error of the mean. (TIFF) [file pbio.3001530.s002.tiff]

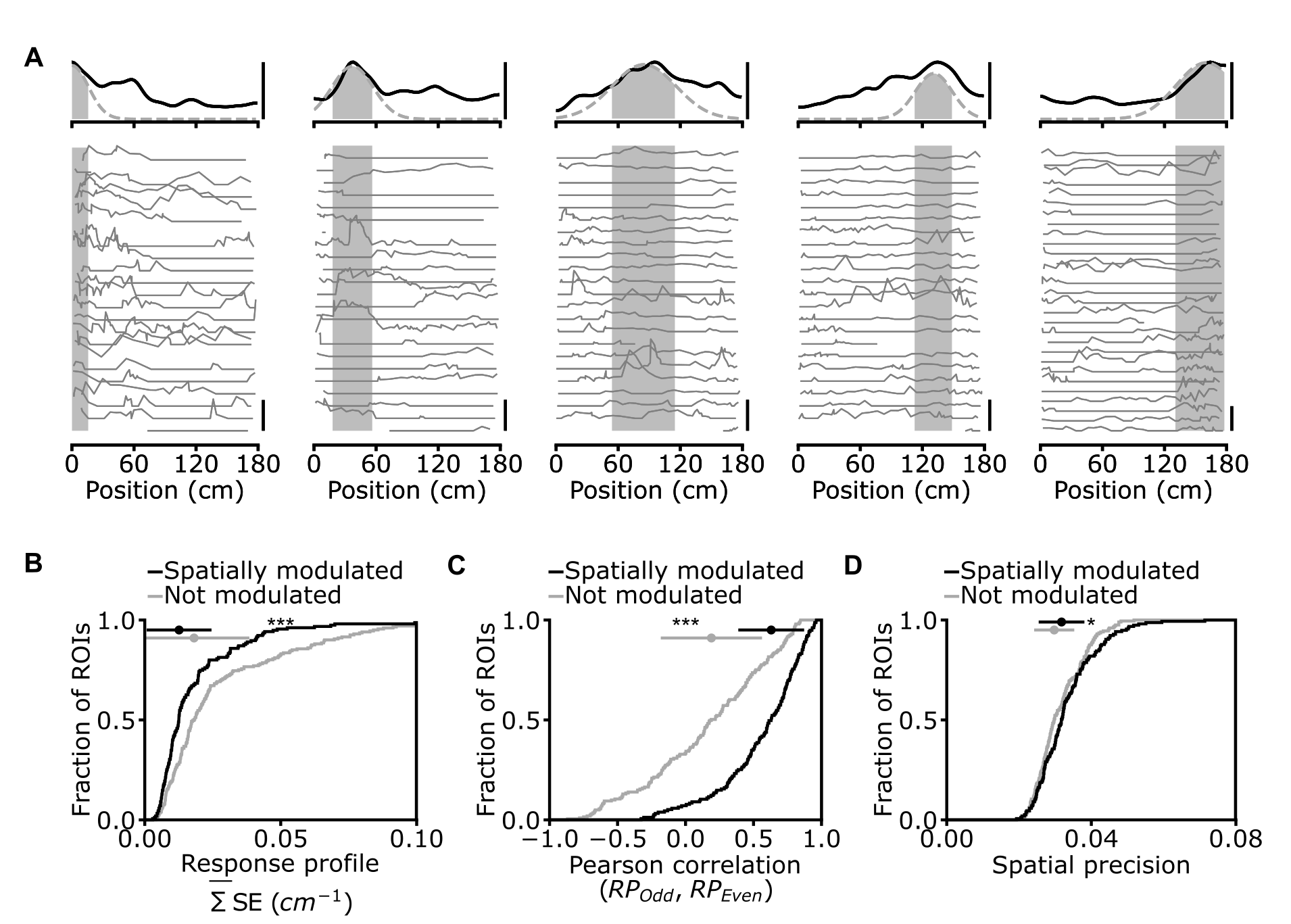

Supplement: S3 Fig — (A) Representative traces showing calcium signals for 5 astrocytic ROIs encoding spatial information shown in Fig 1E. Top: Solid black lines indicate the average astrocytic calcium response across runs as a function of spatial position, and the dashed gray lines indicate response field Gaussian fitting function. Bottom: Solid gray lines indicate normalized calcium event traces as a function of position in the virtual corridor for individual runs. Filled gray areas indicate response field width. (B) Cumulative distribution of the mean SE of the response profile in astrocytic ROIs (median ± MAD 1.3E-2 ± 1.2E-2 cm−1, N = 155 out of 356 total ROIs, for ROIs with reliable spatial information, black; 1.8E-2 ± 2.0E-2 cm−1, N = 201 out of 356 total ROIs, for not modulated ROIs, gray: p = 1E-5, Kolmogorov–Smirnov test). (C) Cumulative distribution of Pearson correlation values between astrocytic response profiles in even and odd trials (median ± MAD 0.63 ± 0.24, N = 155 out of 356 total ROIs for ROIs with reliable spatial information, black; 0.19 ± 0.37, N = 201 out of 356 total ROIs, for not modulated ROIs, gray; p = 5E-14, Kolmogorov–Smirnov test). (D) Cumulative distribution of the spatial precision index of the response field of astrocytic ROIs (black: median ± MAD 3.2E-2 ± 0.6E-2, N = 155 out of 356 total ROIs, for ROIs with reliable spatial information; gray: 3.0E-2 ± 0.5E-2 cm−1, N = 201 out of 356 total ROIs, for not modulated ROIs: p = 3.8E-2, Kolmogorov–Smirnov test). In all panels, data from 7 imaging sessions in 3 animals. The data presented in this figure can be found in S2 Data. MAD, median absolute deviation; ROI, region of interest; SE, standard error. (TIFF) [file pbio.3001530.s003.tiff]

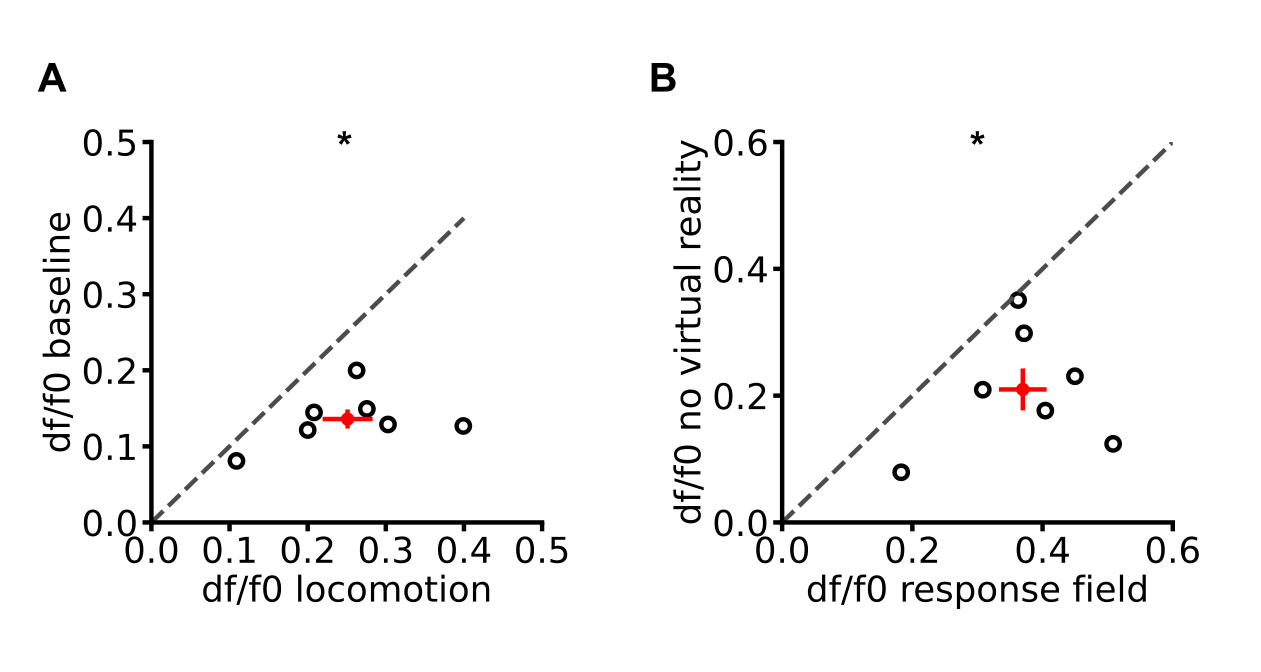

Supplement: S4 Fig — (A) Scatterplot of the average ΔF/F0 of astrocytic ROIs during baseline (mouse speed ≤ 1 cm/s) versus during locomotion (mouse speed > 1cm/s). Under both conditions, the mouse was immersed in the virtual reality. Black open dots represent averages of each imaging session. The red cross shows the mean ± SEM of plotted data (mean ΔF/F0 during baseline 0.14 ± 0.01; mean ΔF/F0 during locomotion 0.25 ± 0.03; N = 356 ROIs; p = 0.016 Wilcoxon signed rank test). (B) Same as in (A) but for ΔF/F0 values measured in astrocytic ROIs encoding reliable spatial information when the mouse was not exposed to the visual stimulation of the virtual reality (during intertrial intervals) versus when the mouse was passing through each ROIs’ response fields (mean ΔF/F0 during without visual stimulation 0.21 ± 0.03; mean ΔF/F0 inside the response field 0.37 ± 0.04; N = 155 out of 356 total ROIs; p = 0.016 Wilcoxon signed rank test). Data in (A, B) from 7 imaging sessions in 3 animals. The data presented in this figure can be found in S2 Data. ROI, region of interest; SEM, standard error of the mean. (TIFF) [file pbio.3001530.s004.tiff]

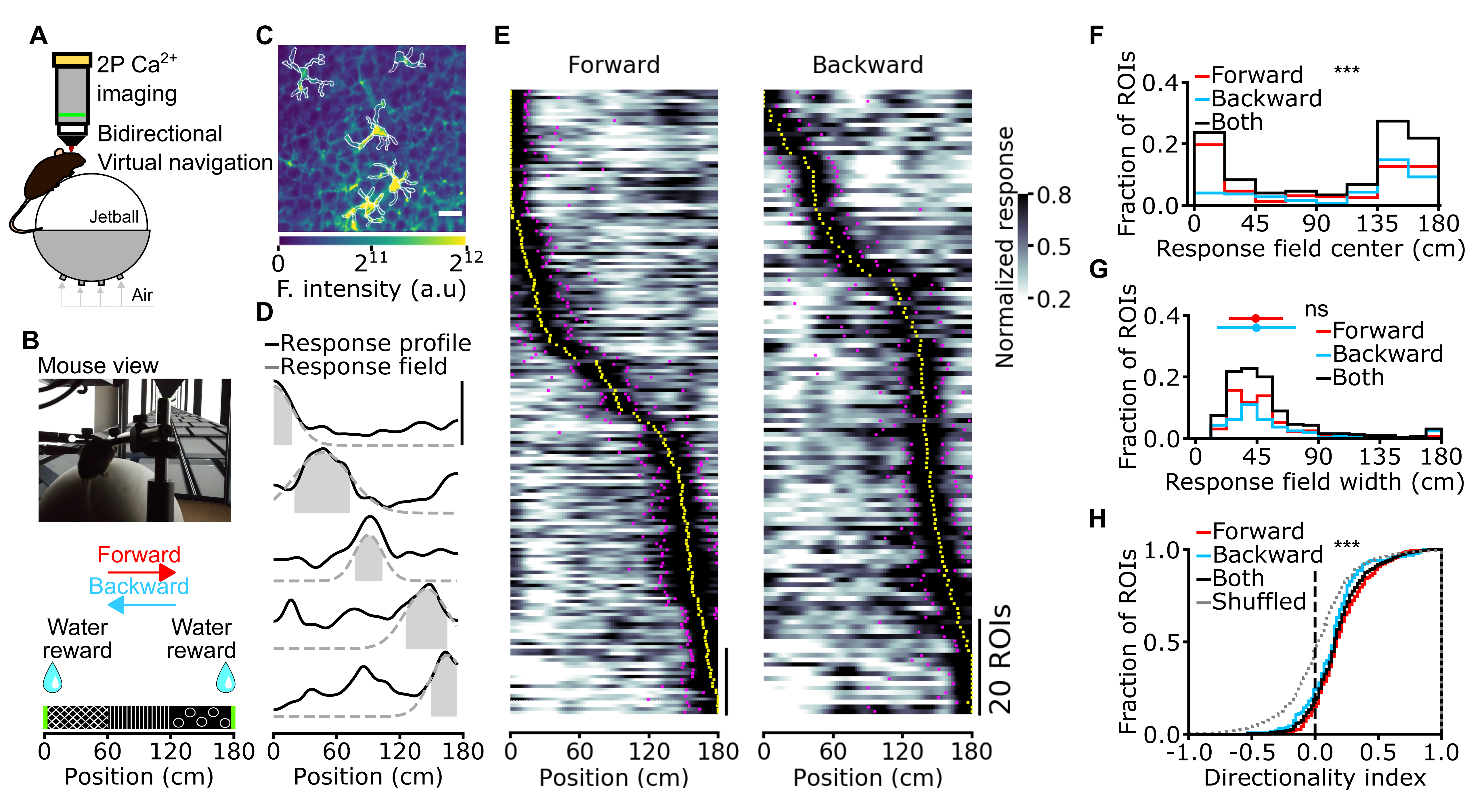

Supplement: S5 Fig — (A) Two-photon functional imaging of CA1 astrocytes is performed during bidirectional virtual navigation. (B) Head-restrained mice run on an air-suspended spherical treadmill in a linear virtual track in both forward and backward directions. Water rewards are delivered at either end of the virtual corridor. (C) Median projection of GCaMP6f-labeled astrocytes in the CA1 pyramidal layer. White lines indicate segmented ROIs. Scale bar: 20 μm. (D) Calcium signals for 5 representative astrocytic ROIs reliably encoding spatial information across the corridor length. Solid black lines indicate the average astrocytic calcium response across trials as a function of spatial position. Dashed gray lines and filled gray areas indicate the Gaussian fitting function and the response field width (see Methods), respectively. (E) Normalized astrocytic calcium responses as a function of position for astrocytic ROIs with reliable spatial information. Trials are divided according to running direction (forward and backward). For forward trials, informative ROIs are N = 192 out of 648 total ROIs, mean ± SD: 29 ± 13%; for backward trials, informative ROIs are N = 133 out of 648 ROIs, mean ± SD: 20 ± 13%, p = 0.09, Wilcoxon signed rank test. Scale bar: 20 ROIs. Yellow dots indicate the center position of the response field, and the magenta dots indicate the width of the field response. (F) Distributions of astrocytic response field position for forward and backward running direction. Median ± MAD 93 ± 66 cm, N = 192 out of 648 total ROIs for the forward direction; 138 ± 47 cm N = 133 out of 648 total ROIs for the backward direction; p = 9E-7, Kolmogorov–Smirnov test). (G) Distributions of response field width for the forward and backward running direction (response field width, 44 ± 19 cm, N = 192 out of 648 total ROIs for the for forward direction; response field width, 44 ± 28 cm, N = 133 out of 648 total ROIs for the backward direction; p = 0.34, Wilcoxon rank sums test). (H) DI for forw [file pbio.3001530.s005.tiff]

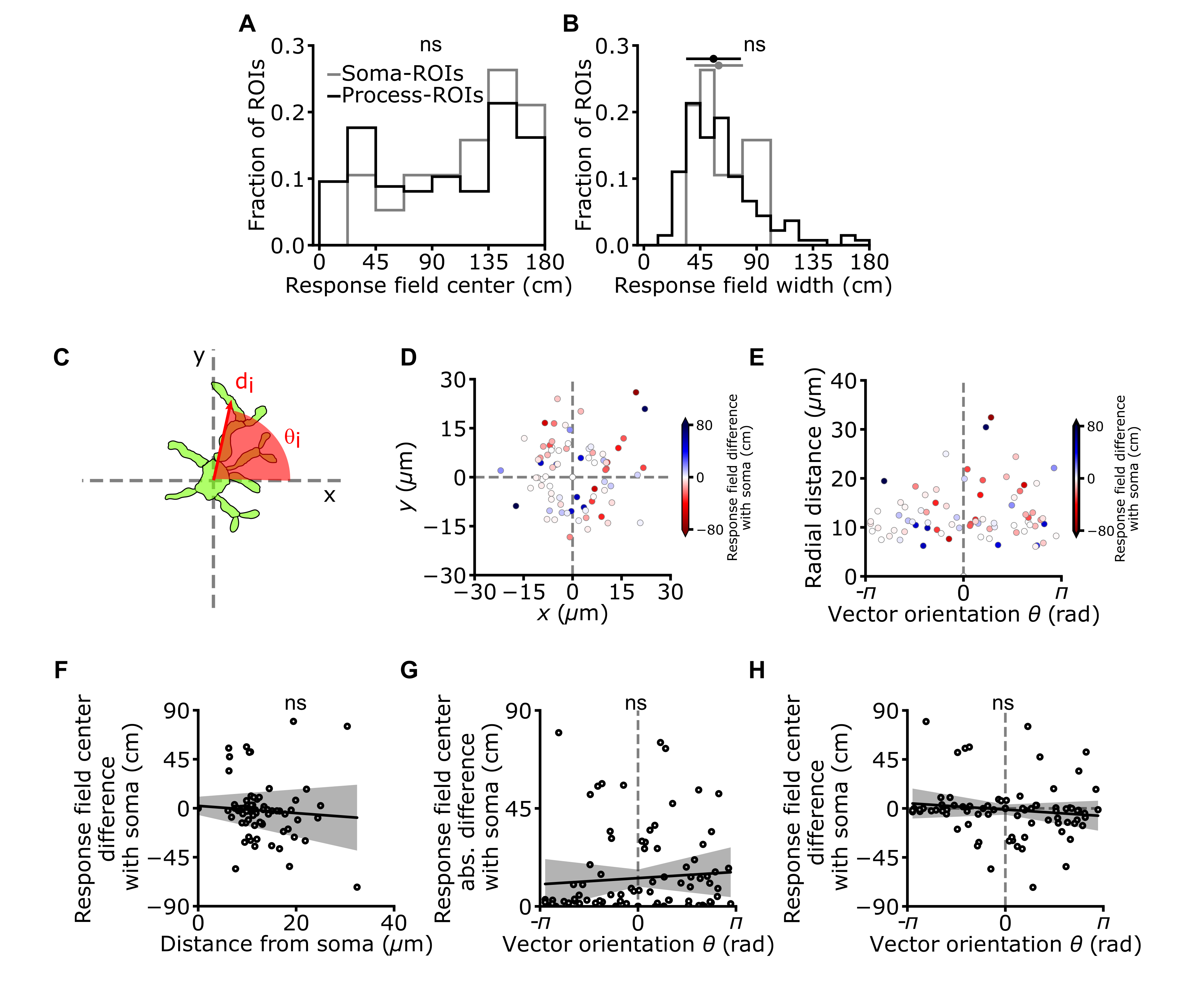

Supplement: S6 Fig — (A) Distribution of field position for soma ROIs and process ROIs (p = 0.36, Kolmogorov–Smirnov test). (B) Distribution of response field width for astrocytic soma ROIs and process ROIs (median width for soma ROIs: 60 ± 19 cm; median width for process ROIs: 56 ± 22 cm, p = 0.36, Wilcoxon rank sums test). (C) For each pair of ROIs within a given astrocyte, the distance (d) between the centers of 2 ROIs and the angle between the line connecting the 2 ROI centers and the x-axis are calculated. Only astrocytes showing significant spatial modulation in the soma and at least 1 process were used for this analysis. (D, E) Difference in field position of a process with respect to the field position of its corresponding soma, expressed as function of Cartesian (D) and polar (E) coordinates of the ROI centers. (F) Difference in response field position of a process with respect to the field position of its corresponding soma as a function of the process distance from cell soma (R2 = 0.01, p = 3.3E-1, Wald test, data from 19 cells from 7 imaging sessions on 3 animals). (G, H) Absolute value (G) or signed (H) difference in response field position of a process ROI with respect to the field position of its corresponding soma as a function of the process angular coordinate (absolute value of difference in response field R2 = 0.01, p = 4.8E-1, Wald test; signed value of difference in response field R2 = 0.01, p = 4.1E-1, Wald test, data from 19 cells from 7 imaging sessions on 3 animals). The data presented in this figure can be found in S2 Data. ROI, region of interest. (TIFF) [file pbio.3001530.s006.tiff]

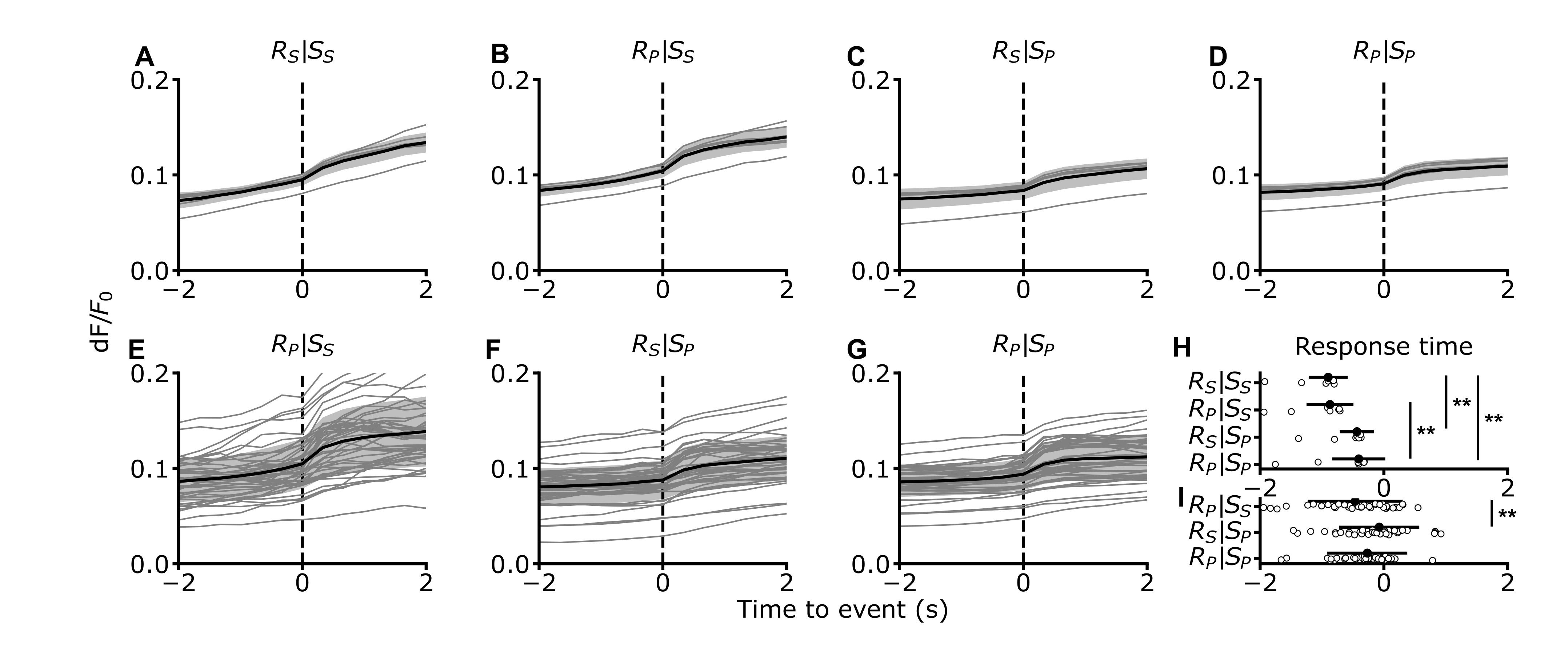

Supplement: S7 Fig — (A) Event triggered average of astrocytic calcium responses. Calcium responses of putative receiver (R) ROIs are aligned to calcium events of putative source (S) ROIs according to anatomic identities of ROIs (e.g., somatic receiver ROIs and somatic source ROIs). Data from 7 imaging sessions in 3 animals. Black line indicates the mean, and shaded area the standard deviation. (B–D) Same as in (A) for pairs of process receiver and somatic source (B), somatic receiver and process source (C), and process receiver and process source (D). (E–G) Same as in (B-D) but for pairs of ROIs belonging to the same astrocyte (N = 46 astrocytes from 7 imaging sessions in 3 animals). (H) Response time (see Methods) for signals shown in (A-D). p = 6E-4, Friedman test with Nemenyi post hoc correction. (I) Response time for signals shown in (E-G). p = 7E-3, Friedman test with Nemenyi post hoc correction. The data presented in this figure can be found in S2 Data. ROI, region of interest. (TIFF) [file pbio.3001530.s007.tiff]

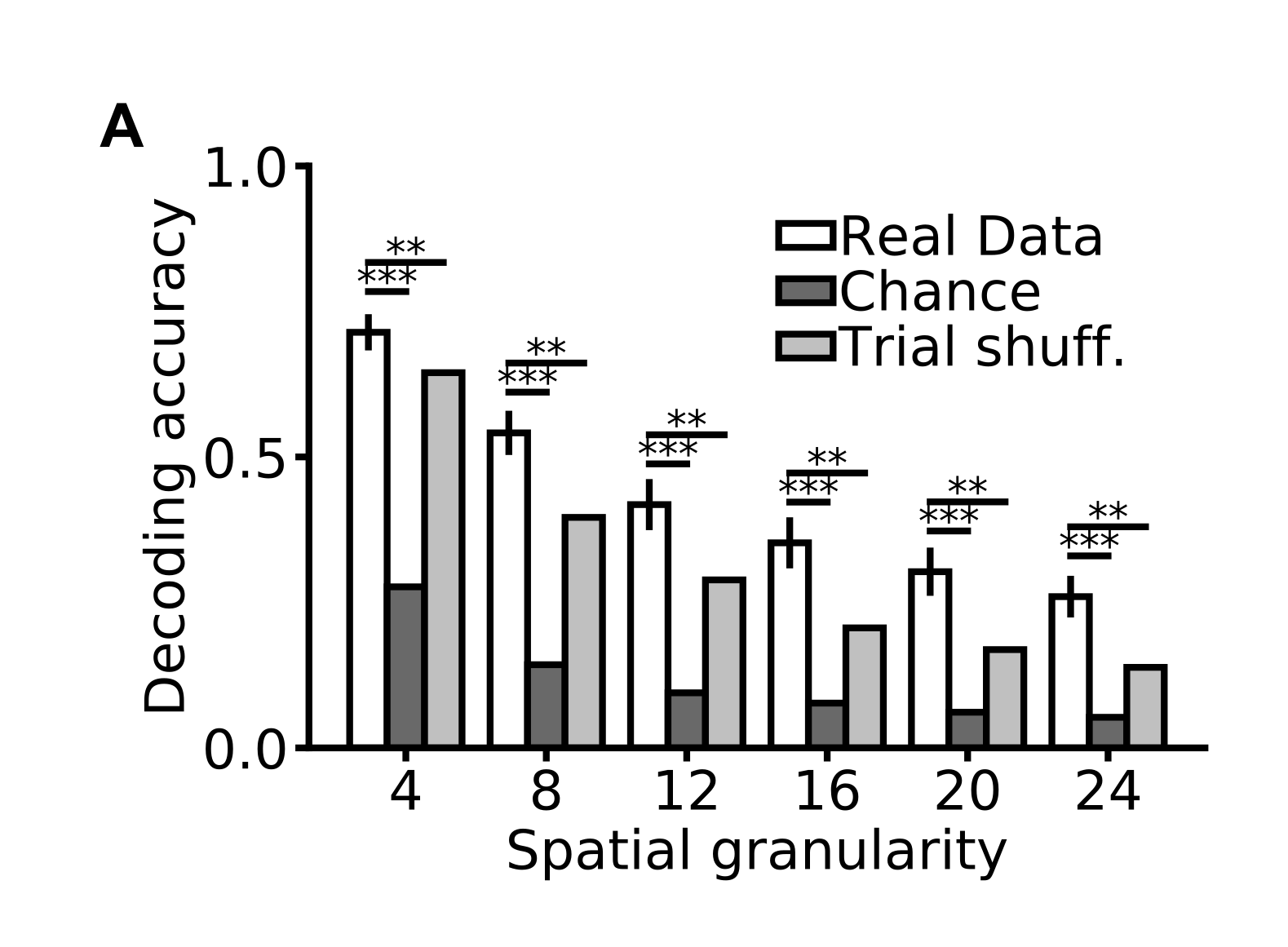

Supplement: S8 Fig — (A) Decoding accuracy as a function of spatial granularity on real (white), chance (dark gray), and trial-shuffled (gray) data (see Methods). Data are presented as mean ± SEM from 7 imaging sessions on 3 animals; see also S2 Table. The data presented in this figure can be found in S2 Data. SEM, standard error of the mean. (TIFF) [file pbio.3001530.s008.tiff]

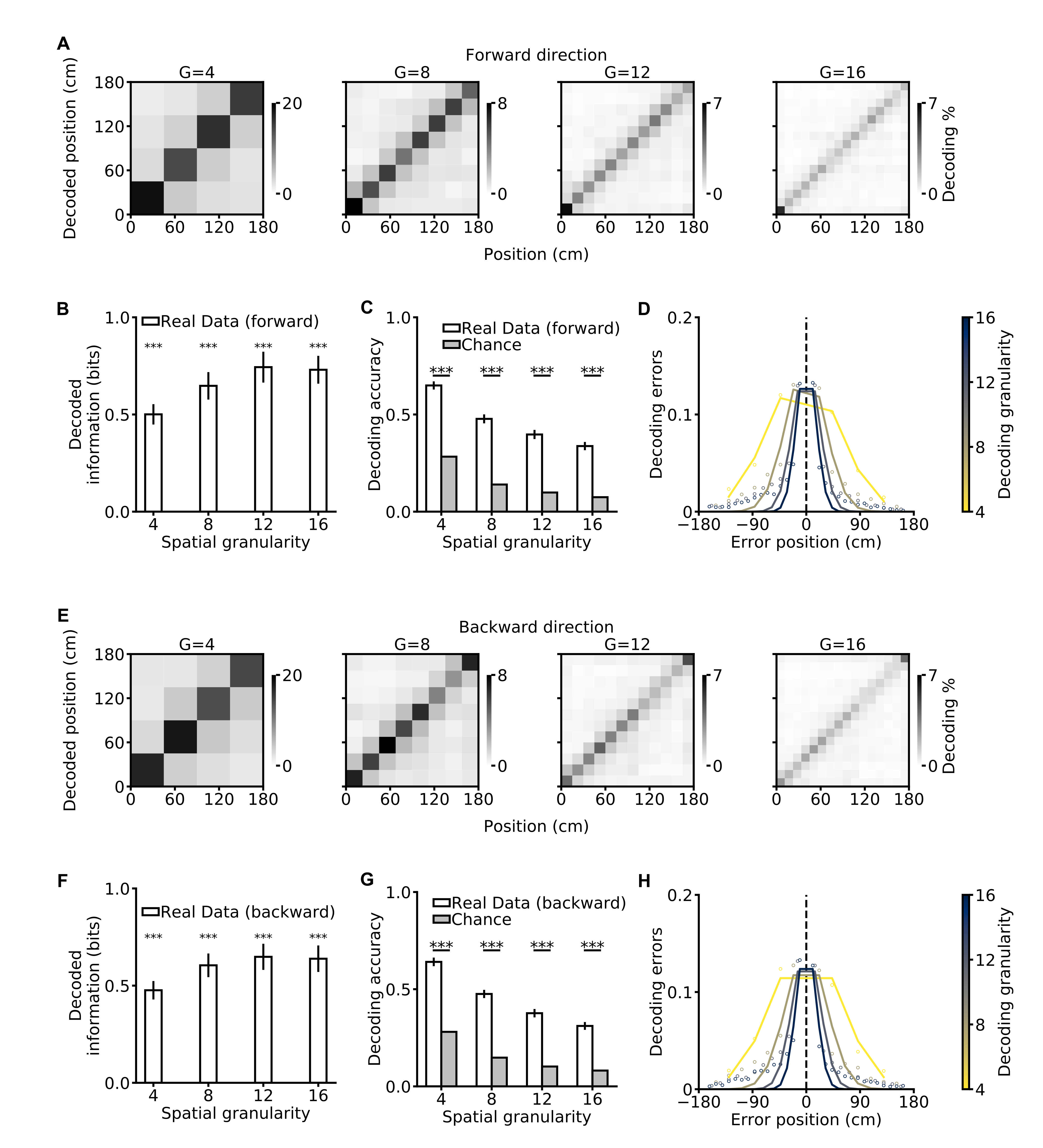

Supplement: S9 Fig — (A) Confusion matrices of an SVM classifier for different spatial granularities (G = 4, 8, 12, 16) for trials in which the mouse was running in the forward direction (forward). The actual position of the animal is shown on the x-axis, the decoded position on the y-axis. Gray scale indicates the number of events in each matrix element. (B) Decoded information as a function of spatial granularity on real (white) and chance (gray) data for forward trials. (C) Decoding accuracy as a function of spatial granularity. (D) Decoding error as a function of the error position within the confusion matrix for forward trials. The color code indicates spatial granularity. In panels (A–D), data from 15 imaging sessions in 4 animals. (E–H) Same as in (A–D) for trials in the backward direction. Data from 17 imaging sessions in 4 animals. In (B, C, F, G), data are presented as mean ± SEM. See also S6 Table. The data presented in this figure can be found in S3 Data. SEM, standard error of the mean; SVM, support vector machine. (TIFF) [file pbio.3001530.s009.tiff]

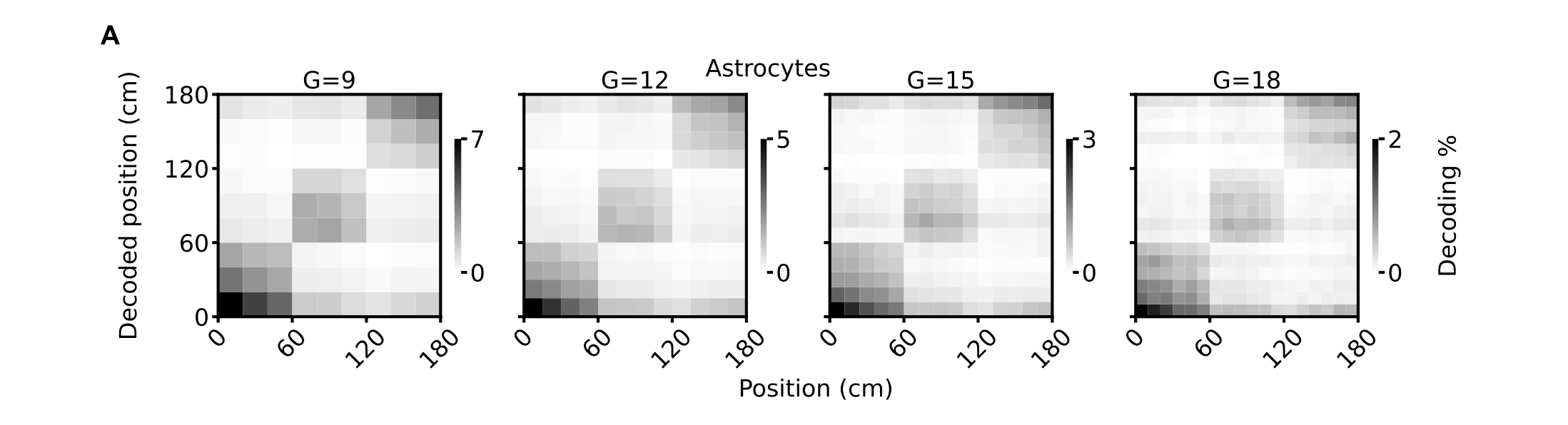

Supplement: S10 Fig — (A) Confusion matrices of an SVM classifier decoding the mouse’s position using population vectors data comprising astrocytic ROIs in which position was shuffled within visual cues. Shuffling position within visual cues decoupled spatial information encoded in the population vector from the information related to visual cues identity (see Methods). The true position of the animal is shown on the x-axis and the decoded position on the y-axis. Gray scale indicates the percentage of occurrence of each matrix element (Decoding). Results are shown for various spatial granularities (G = 9, 12, 15, 18). In all panels, data from 500 permutations on 7 imaging sessions in 3 animals are shown. The data presented in this figure can be found in S3 Data. ROI, region of interest; SVM, support vector machine. (TIFF) [file pbio.3001530.s010.tiff]

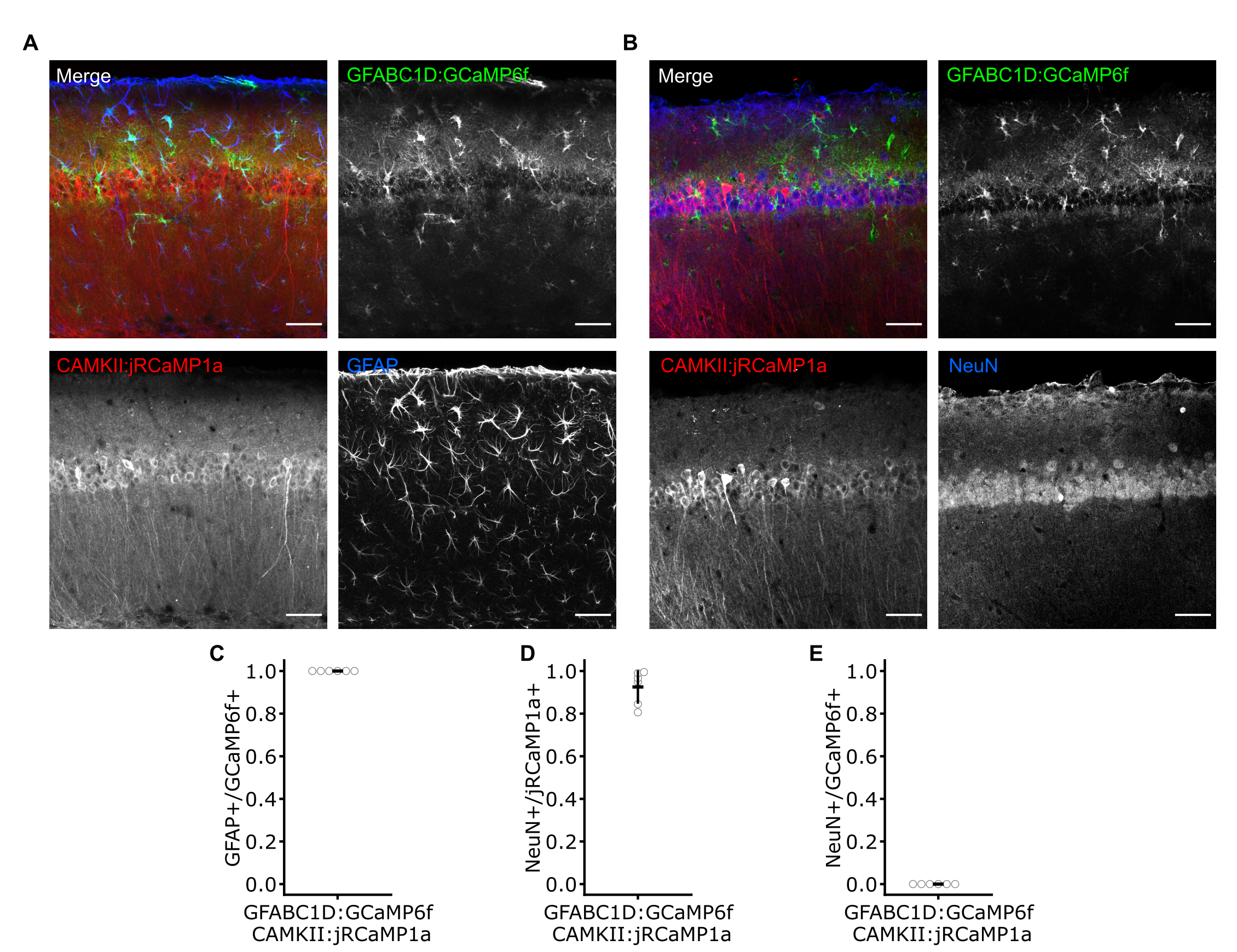

Supplement: S11 Fig — (A, B) Representative images of hippocampal CA1 areas from animals transduced with AAV5 pZac2.1 gfaABC1D-cyto-GCaMP6f and AAV1/2 pAAV CAMKII-jRCaMP1a implanted with a chronic optical window. Images are maximum intensity projection of confocal z-stacks (9 planes, 2 μm/step) from brain slices stained either with anti-GFAP (A) or an anti-NeuN primary antibody (B). In both cases, counterstaining was performed with an Alexa-647 conjugated secondary antibody. (C) Related to (A): Fraction of GCaMP6f-expressing cells immunolabeled for GFAP (100 ± 0%, out of a total of 71 GCaMP6f-expressing cells from N = 6 sections from 3 mice). (D) Related to (B): fraction of jRCaMP1a-expressing cells immunolabeled for NeuN (93 ± 8%, out of a total of 985 jRCaMP1a-expressing cells from N = 6 sections from 3 mice). (E) Same as in (D) but for GCaMP6f-expressing cells (0 ± 0%, out of a total of 50 GCaMP6f-expressing cells, from N = 6 sections from 3 mice). Scale bars in A and B: 50 μm. The data presented in this figure can be found in S3 Data. GFAP, glial fibrillary acidic protein. (TIFF) [file pbio.3001530.s011.tiff]

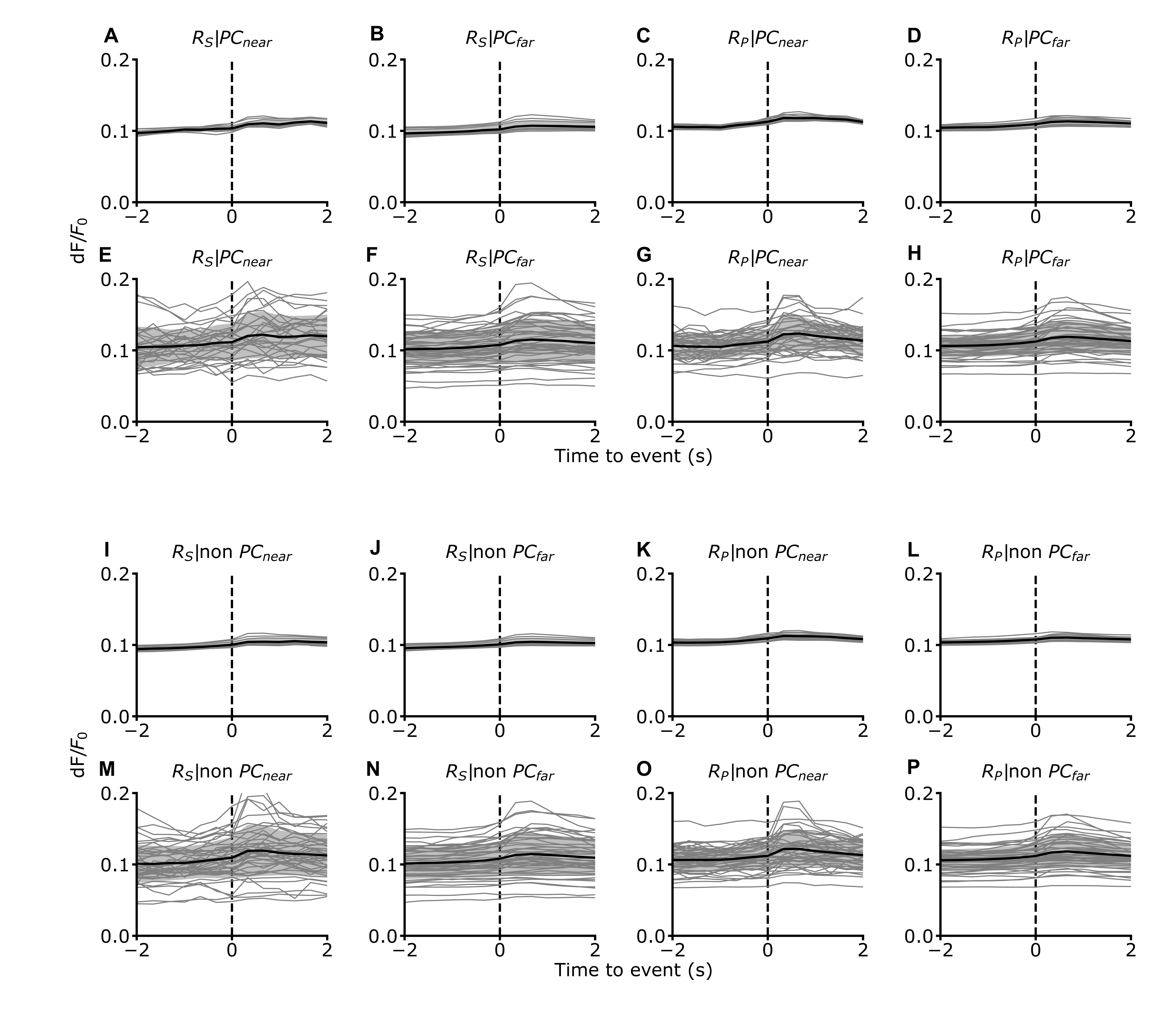

Supplement: S12 Fig — (A–D) Event triggered average of astrocytic calcium responses. Calcium responses of putative receiver (R) ROIs are aligned to calcium events of neuronal PCs. Astrocytic receiver ROIs could be in the soma (s) or processes (p). Neuronal cells were classified as being close (≤15 μm) or far (>15 μm) from astrocytic receiver ROIs. Data from 11 imaging sessions in 7 animals. The black line indicates the mean, the shaded area the standard deviation. (E, F) Same as in (A–D) but for receiver ROIs belonging to the same astrocyte (N = 23 cells from 11 imaging sessions in 7 animals). (I–L) Same as in (A–D) but calcium responses of putative receiver (R) ROIs are aligned to calcium events of nonspatially informative cells (non-PC). Data from 11 imaging sessions in 7 animals. (M–P) Same as in (I–L) but for receiver ROIs belonging to the same astrocyte (N = 48 astrocytes from 11 imaging sessions in 7 animals). The data presented in this figure can be found in S3 Data. PC, place cell; ROI, region of interest. (TIFF) [file pbio.3001530.s012.tiff]

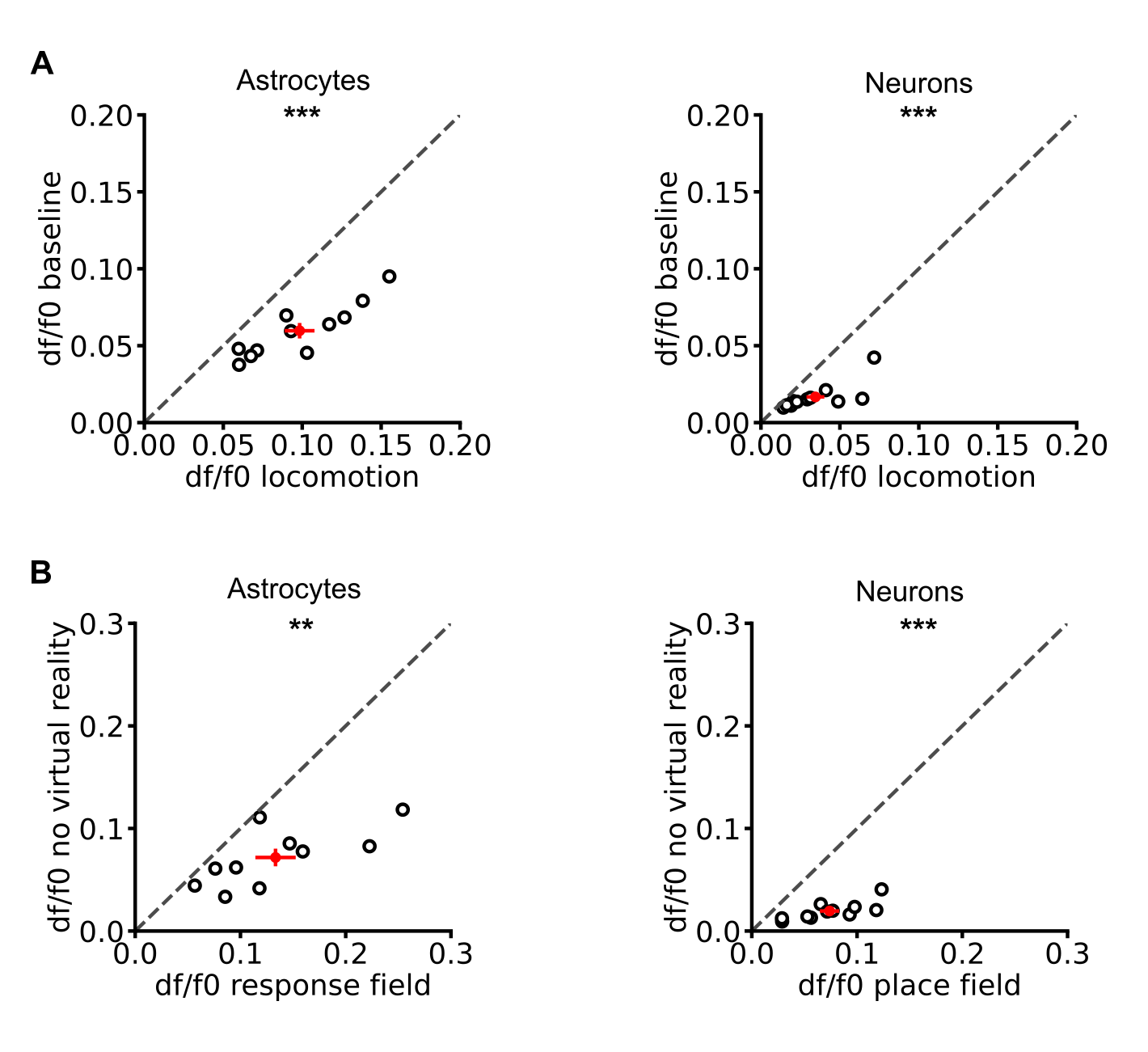

Supplement: S13 Fig — (A) Scatterplot of the average ΔF/F0 of astrocytic ROIs during baseline (mouse speed ≤ 1 cm/s) versus during locomotion (mouse speed > 1 cm/s). Under both conditions, the mouse was immersed in the virtual reality. Black open dots show averages of each imaging session. The red cross shows the mean ± SEM of plotted data. Average ΔF/F0 values were measured in astrocytic ROIs (left; mean ΔF/F0 during baseline 0.06 ± 0.01; mean ΔF/F0 during locomotion 0.10 ± 0.01, N = 341 ROIs; p = 9.8E-4 Wilcoxon signed rank test) and neuronal ROIs (right; mean ΔF/F0 during baseline 0.017 ± 0.003; mean ΔF/F0 during locomotion 0.03 ± 0.01, N = 870 ROIs; p = 9.8E-4 Wilcoxon signed rank test) recorded from mice co-injected with AAV5 pZac2.1 gfaABC1D-cyto-GCaMP6f and AAV1/2 pAAV-CAMKII-jRCaMP1a. (B) Same as in (A) but for ΔF/F0 values measured in ROIs encoding reliable spatial information when the mouse was not exposed to the visual stimulation of the virtual reality versus when the mouse was passing through each ROIs’ response fields. Astrocytic ROIs, left, (mean ΔF/F0 without visual stimulation 0.07 ± 0.01; mean ΔF/F0 inside the response field 0.13 ± 0.02; p = 0.016 Wilcoxon signed rank test), neuronal ROIs, right, (mean ΔF/F0 without visual stimulation 0.020 ± 0.002; mean ΔF/F0 inside the response field 0.07 ± 0.01; p = 0.016 Wilcoxon signed rank test). Data in (A, B) are presented as mean ± SEM and come from 11 imaging sessions in 7 animals. The data presented in this figure can be found in S3 Data. ROI, region of interest; SEM, standard error of the mean. (TIFF) [file pbio.3001530.s013.tiff]

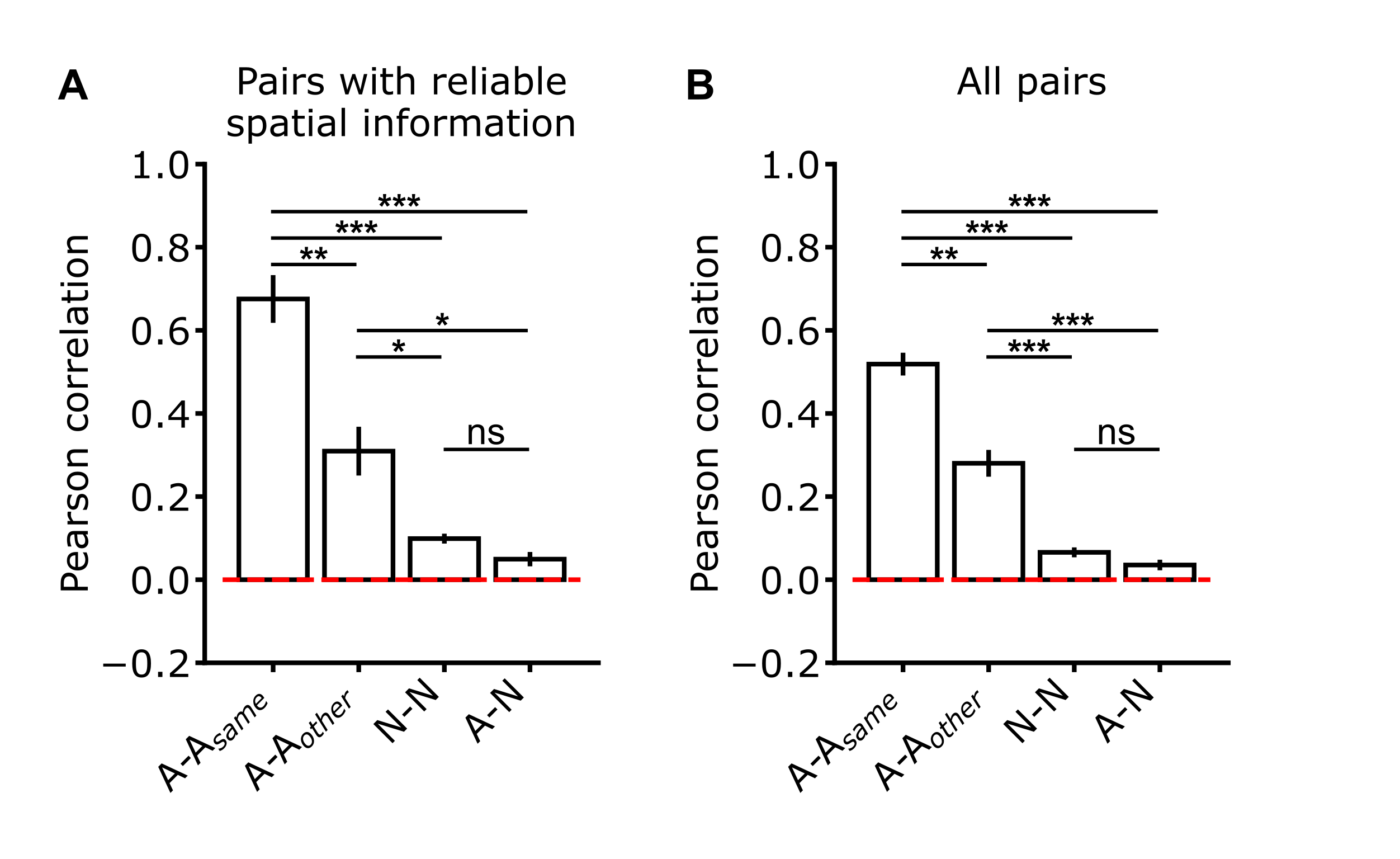

Supplement: S14 Fig — (A, B) Pearson correlation for different pairs of ROIs. Pairs were composed either of two astrocytic ROIs belonging to the same astrocyte (A-Asame), two astrocytic ROIs belonging to the different astrocytes (A-Aother), two neuronal ROIs (N-N), or one astrocytic and one neuronal ROI (A-N). Red line indicates the zero correlation level. In (A), only results for ROI pairs with reliable spatial information are reported (p = 5.2E-3, p = 6.5E-4, p = 9.4E-4, p = 1.5E-2, p = 1.5E-2, p = 9.9E-2 for A-Asame versus A-Aother, A-Asame versus N-N, A-Asame versus A-N, A-Aother versus N-N, A-Aother versus A-N, N-N versus A-N, respectively. Wilcoxon rank sums test with Bonferroni post hoc correction). In (B), results for all possible pairs are displayed (p = 2.6E-3, p = 4.3E-4, p = 4.3E-4, p = 8.6E-4, p = 8.6E-4, p = 1.4E-1 for A-Asame versus A-Aother, A-Asame versus N-N, A-Asame versus A-N, A-Aother versus N-N, A-Aother versus A-N, N-N versus A-N, respectively. Wilcoxon rank sums test with Bonferroni post hoc correction). Data are presented as mean ± SEM from 11 imaging sessions on 7 animals. Data from astrocytic recording comprises 36 cells in which there was significant spatial modulation in at least 1 ROI. The data presented in this figure can be found in S4 Data. ROI, region of interest; SEM, standard error of the mean. (TIFF) [file pbio.3001530.s014.tiff]

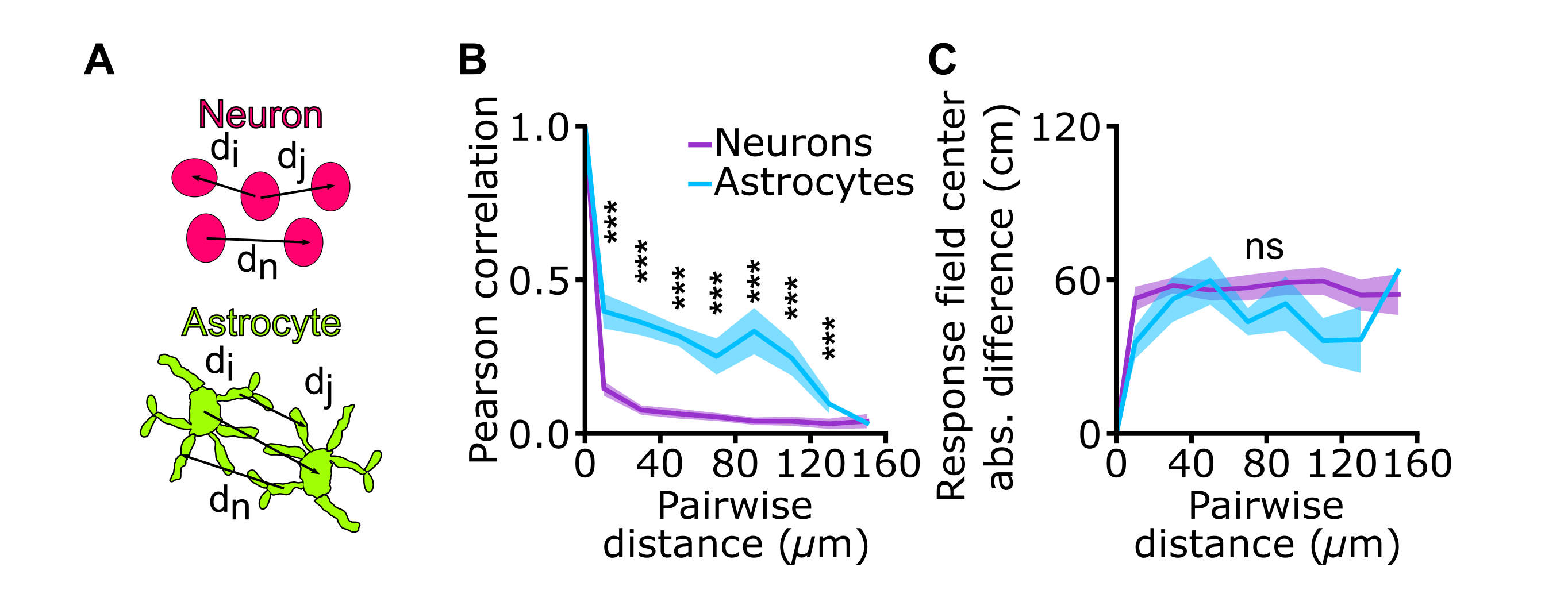

Supplement: S15 Fig — (A) The distance (d) between the centers 2 ROIs comprising a pair is computed for all astrocytic (top) and neuronal (bottom) ROIs. (B, C) Pearson correlation (B) and difference between response field position (C) as a function of pairwise distance for pairs of astrocytic ROIs with reliable spatial information (cyan) and pairs of neuronal ROIs with reliable spatial information (purple). Data are expressed as mean ± SEM from 11 imaging sessions on 7 animals. (A) p = 8E-4, p = 8E-4, p = 1E-4, p = 1E-3, p = 1E-3, p = 1E-3, p = 8E-4, and p = 2E-1 for 10, 30, 70, 90, 110, 130, and 150 μm pairwise distances, respectively. Two-sample Kolmogorov–Smirnov test with Bonferroni post hoc correction. (B) p = 1, p = 1, p = 0.7, p = 1, p = 1, p = 1, p = 0.2, and p = 0.2 for 10, 30, 70, 90, 110, 130, and 150 μm pairwise distances, respectively. Two-sample Kolmogorov–Smirnov test with Bonferroni post hoc correction. Data from astrocytic recording comprises 36 cells in which there was significant spatial modulation in at least 1 ROI. The data presented in this figure can be found in S5 Data. ROI, region of interest; SEM, standard error of the mean. (TIFF) [file pbio.3001530.s015.tiff]

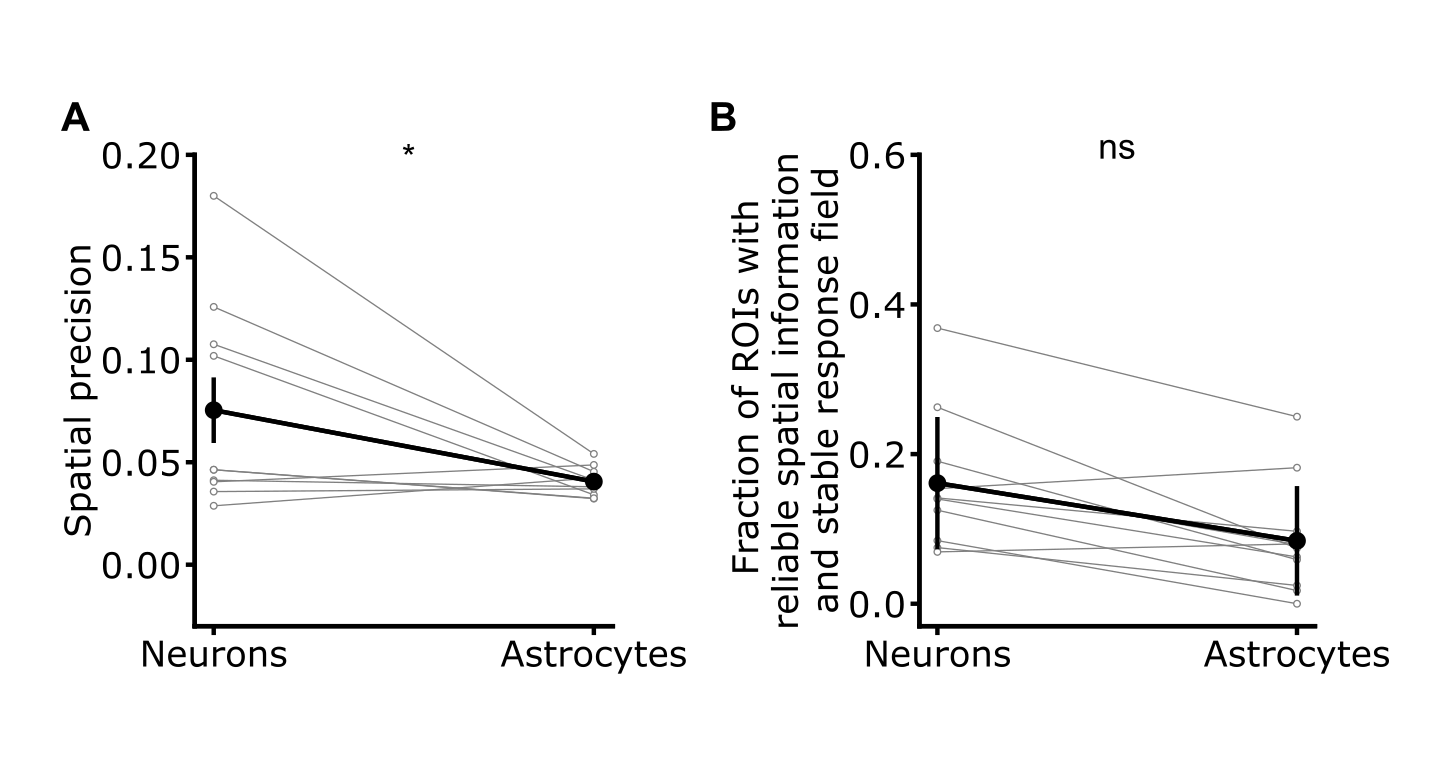

Supplement: S16 Fig — (A) Spatial precision index for simultaneously recorded neuronal and astrocytic response fields (mean ± SEM; neuronal responses 7.5E-2 ± 1.6E-2; astrocytic responses 4.1E-2 ± 0.2E-2; p = 4.6E-2 Wilcoxon signed rank test; data from 11 imaging sessions on 7 animals). (B) Fraction of neuronal and astrocytic ROIs showing reliable spatial information and stable response field (mean ± SD; neurons 0.16 ± 0.09; astrocytic responses 0.08 ± 0.07; p = 2.9E-1 Wilcoxon signed rank test; data from 11 imaging sessions on 7 animals). The data presented in this figure can be found in S5 Data. ROI, region of interest; SD, standard deviation; SEM, standard error of the mean. (TIFF) [file pbio.3001530.s016.tiff]

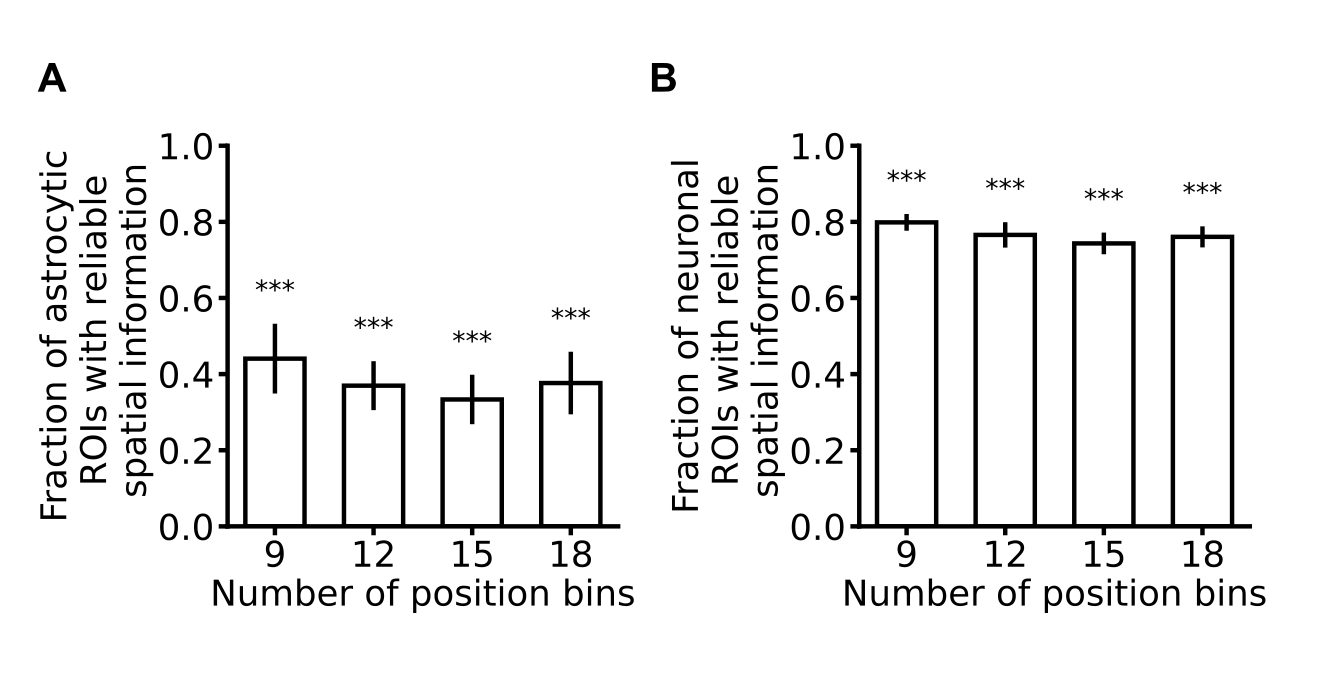

Supplement: S17 Fig — (A, B) Fraction of astrocytic (A) and neuronal (B) ROIs encoding reliable spatial information showing a significant decrease in their information content when position is shuffled within visual cues. Shuffling position within individual visual cues decoupled spatial information encoded in the astrocytic response from the information related to visual cues identity (see Methods). The fraction of ROIs showing significant information loss is shown as function of the number of position bins used to compute mutual information. Data are presented as mean ± SEM from 11 experimental sessions in 7 animals, (N = 76 for astrocytic ROIs, N = 335 for neuronal ROIs, binomial test, see S8 Table). The data presented in this figure can be found in S5 Data. ROI, region of interest; SEM, standard error of the mean. (TIFF) [file pbio.3001530.s017.tiff]

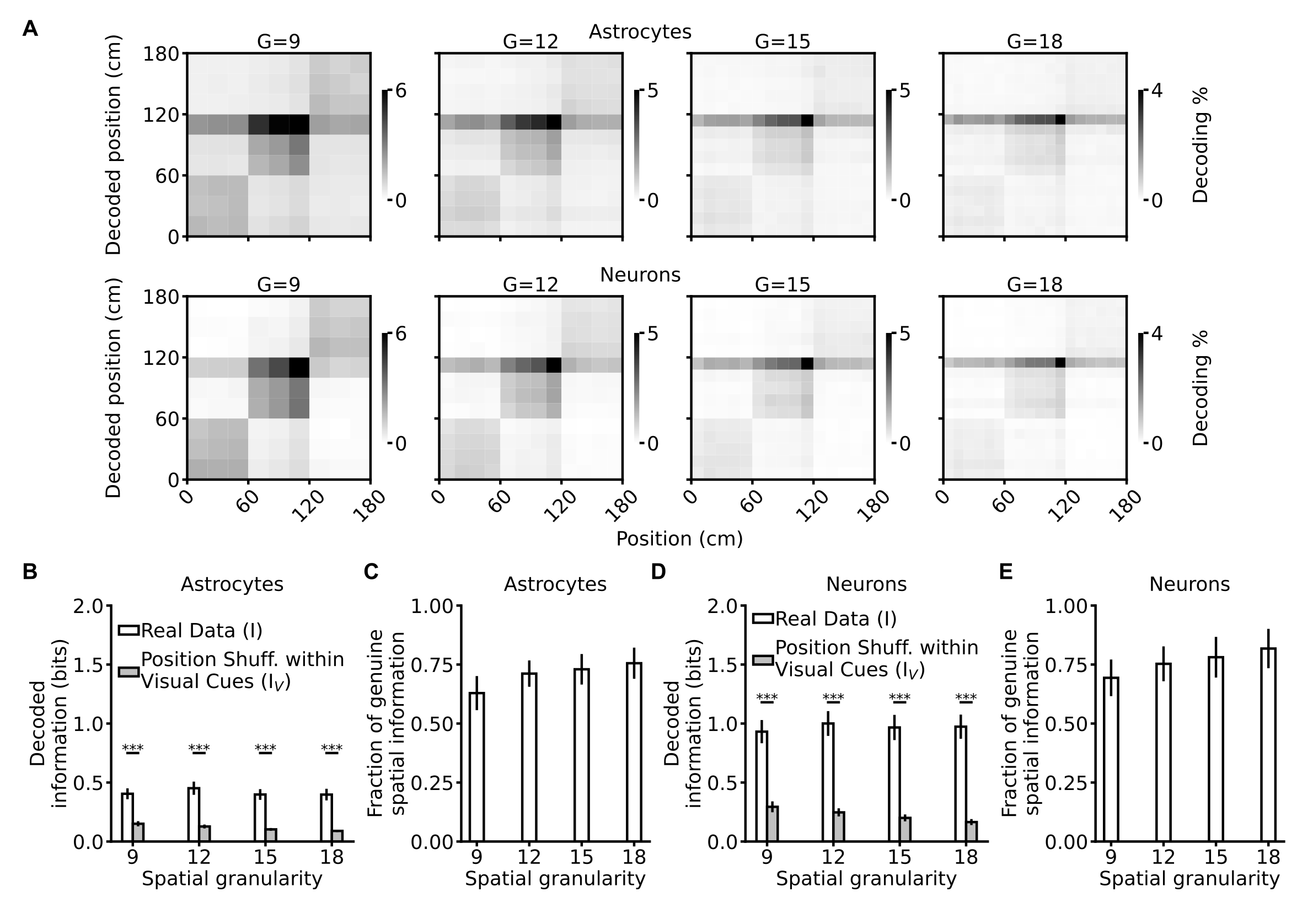

Supplement: S18 Fig — (A) Confusion matrices of an SVM classifier decoding the mouse’s position using population vectors comprising either astrocytic (top) or neuronal (bottom) ROIs in which position was shuffled within visual cues. Shuffling position within visual cues decoupled spatial information encoded in the population vector from the information related to visual cues identity (see Methods). The true position of the animal is shown on the x-axis and the decoded position on the y-axis. Gray scale indicates the percentage of occurrence of each matrix element. Results are shown for various spatial granularities (G = 9, 12, 15, 18). (B) Decoded information from astrocytic population vectors as a function of decoding granularity on real data (white) and for data in which position is shuffled within visual cues (gray, see Methods). (C) Fraction of genuine spatial information in astrocytic population vectors computed shuffling position within individual visual cues. Results are shown as a function of decoding granularity. (D, E) Same as in (B, C) but from population vectors comprising neuronal ROIs. In all panels, data are shown as mean ± SEM and were obtained from 11 imaging sessions in 7 animals (see also S9 Table). The data presented in this figure can be found in S5 Data. ROI, region of interest; SEM, standard error of the mean; SVM, support vector machine. (TIFF) [file pbio.3001530.s018.Tiff]

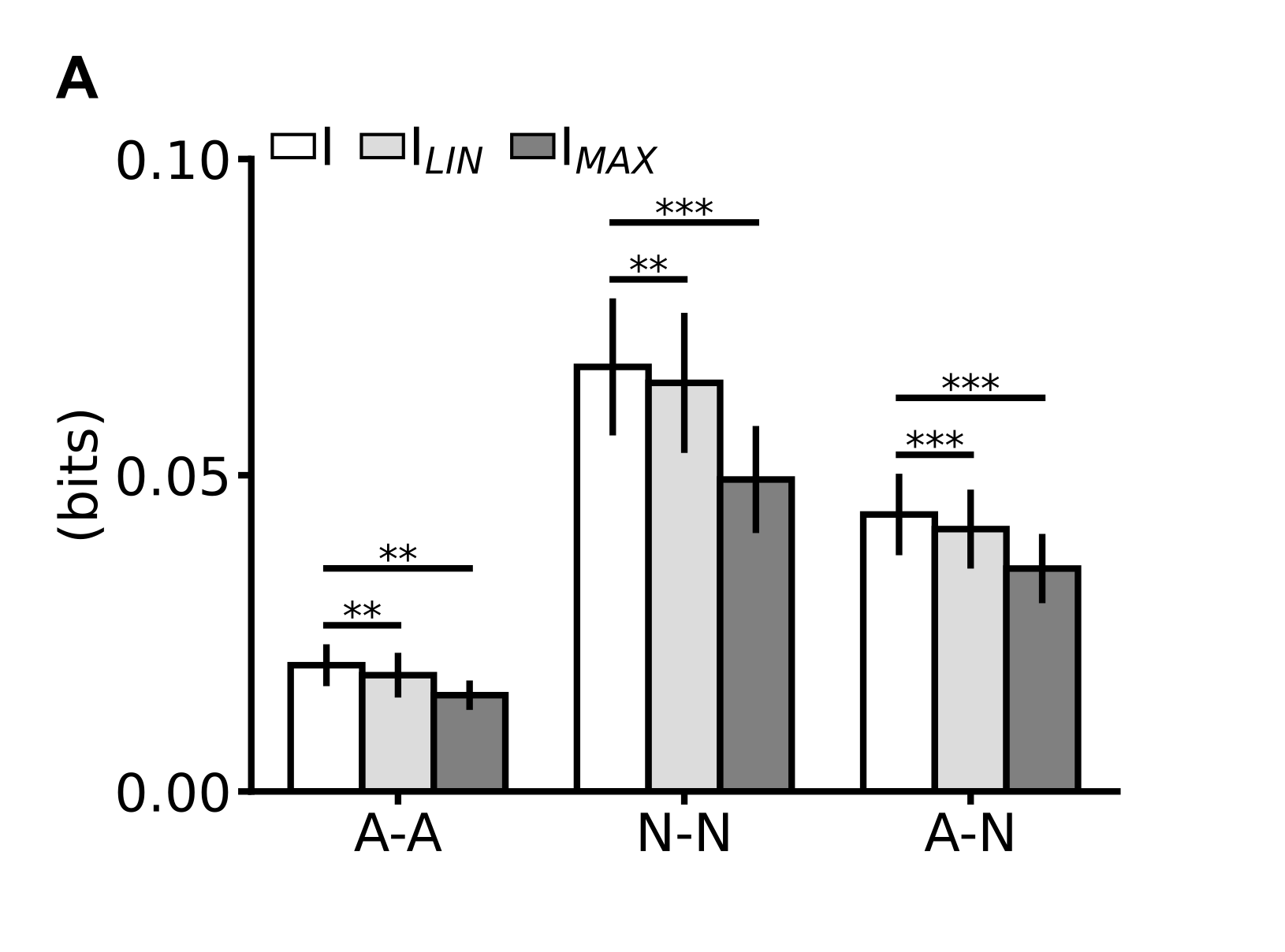

Supplement: S19 Fig — (A) Mutual information about position encoded by pairs of ROIs (I) is shown in comparison to the sum (ILIN) and to the maximum (IMAX) of the information separately encoded by each component of the pair. A-A, pair composed of 2 astrocytic ROIs; N-N, pair composed of 2 neuronal ROIs; A-N, mixed pair composed of one astrocytic and one neuronal ROI. For this analysis, the values of information were computed using the “shuffled” bias correction procedure (see methods) which overcorrects the bias inducing an underestimation of I (I versus ILIN: A-A: p = 1E-2, N-N: p = 7E-3, A-N: p = 1E-3; I versus IMAX: A-A: p = 5E-3, N-N: p = 1E-3, A-N: p = 1E-3, Wilcoxon signed rank test, see also S3 Table). Data are represented as mean ± SEM from 11 imaging sessions in 7 animals. The data presented in this figure can be found in S5 Data. ROI, region of interest, SEM, standard error of the mean. (TIFF) [file pbio.3001530.s019.Tiff]

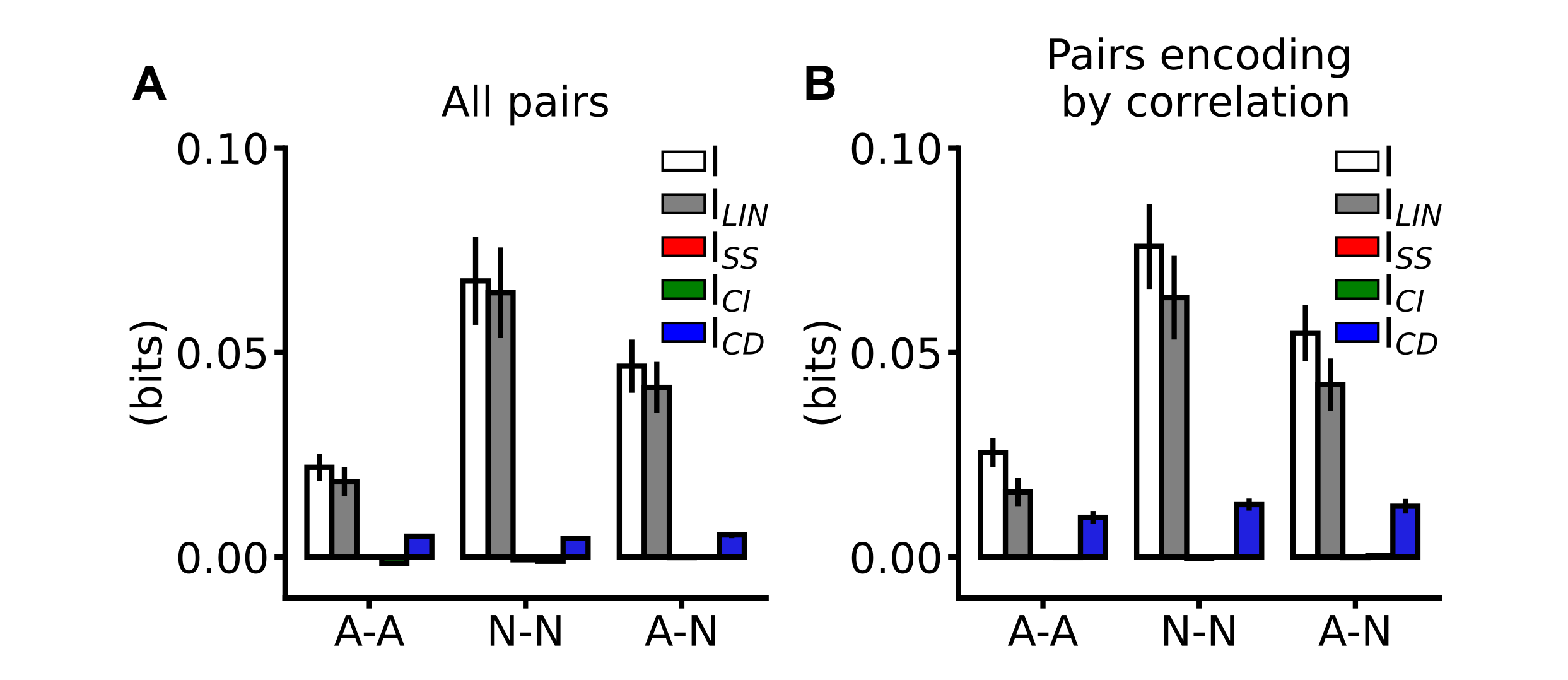

Supplement: S20 Fig — (A, B) Information breakdown for the different types of ROI pairs: 2 astrocytic ROIs (A-A), 2 neuronal ROIs (N-N), or one astrocytic and one neuronal ROI (A-N). Pairs were classified as synergistic (B) based on the value of ΔI (see Methods). I (white) is the mutual information about position encoded by the pair. ILIN (gray) is the sum of the mutual information about position independently encoded in the response of each member of the pair. ISS (red) is the redundant information component quantifying similarity in the responses of the members of the pair. ICI (green) and ICD (blue) quantify the information contribution of correlation independent or dependent on position, respectively. Data are represented as mean ± SEM and were collected in 11 imaging sessions on 7 animals. The data presented in this figure can be found in S5 Data. ROI, region of interest; SEM, standard error of the mean. (TIFF) [file pbio.3001530.s020.tiff]

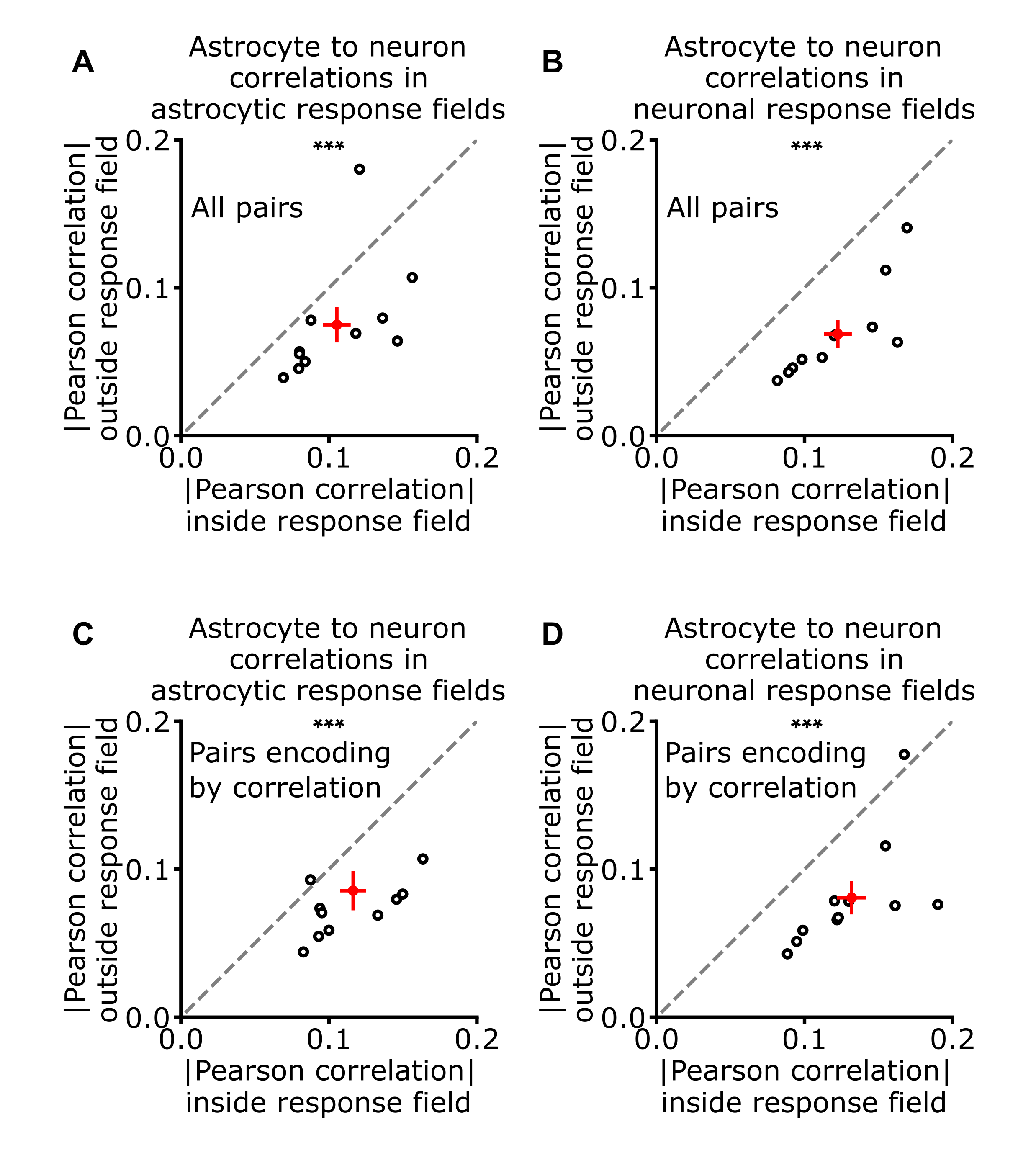

Supplement: S21 Fig — (A–D) Scatterplot of the absolute value of Pearson correlation outside the response field against the absolute value of Pearson correlation inside the response field for pairs comprising one astrocytic and one neuronal ROI. Black open dots show averages of each imaging session, the red cross shows the mean ± SEM (A, B) Correlations were measured for all possible pairs. In (A), correlations are computed with respect to astrocytic response field (mean correlation inside the response field 0.11 ± 0.01; mean correlation outside the response field 0.07 ± 0.01, p = 6.4E-3 Wilcoxon rank sums test). In (B), correlations are computed with respect to neuronal response field (mean correlation inside the response field 0.12 ± 0.01; mean correlation outside the response field 0.07 ± 0.01, p = 1.1E-3 Wilcoxon rank sums test). (C, D) Same as (A, B) but correlations were computed only on synergistic pairs based on the value of ΔI (see Methods, Fig 6, and S11 Fig). In (C), correlations are computed with respect to astrocytic response field (mean correlation inside the response field 0.12 ± 0.01; mean correlation outside the response field 0.09 ± 0.01, p = 7.8E-3 Wilcoxon rank sums test). In (D), correlations are computed with respect to neuronal response field (mean correlation inside the response field 0.13 ± 0.01; mean correlation outside the response field 0.08 ± 0.01, p = 1.8E-3 Wilcoxon rank sums test). For each pair of ROIs, correlations were computed averaging 100 resampling to compensate unbalanced observations inside and outside the response field. Data from 11 imaging sessions on 7 animals. The data presented in this figure can be found in S5 Data. ROI, region of interest; SEM, standard error of the mean. (TIFF) [file pbio.3001530.s021.tiff]

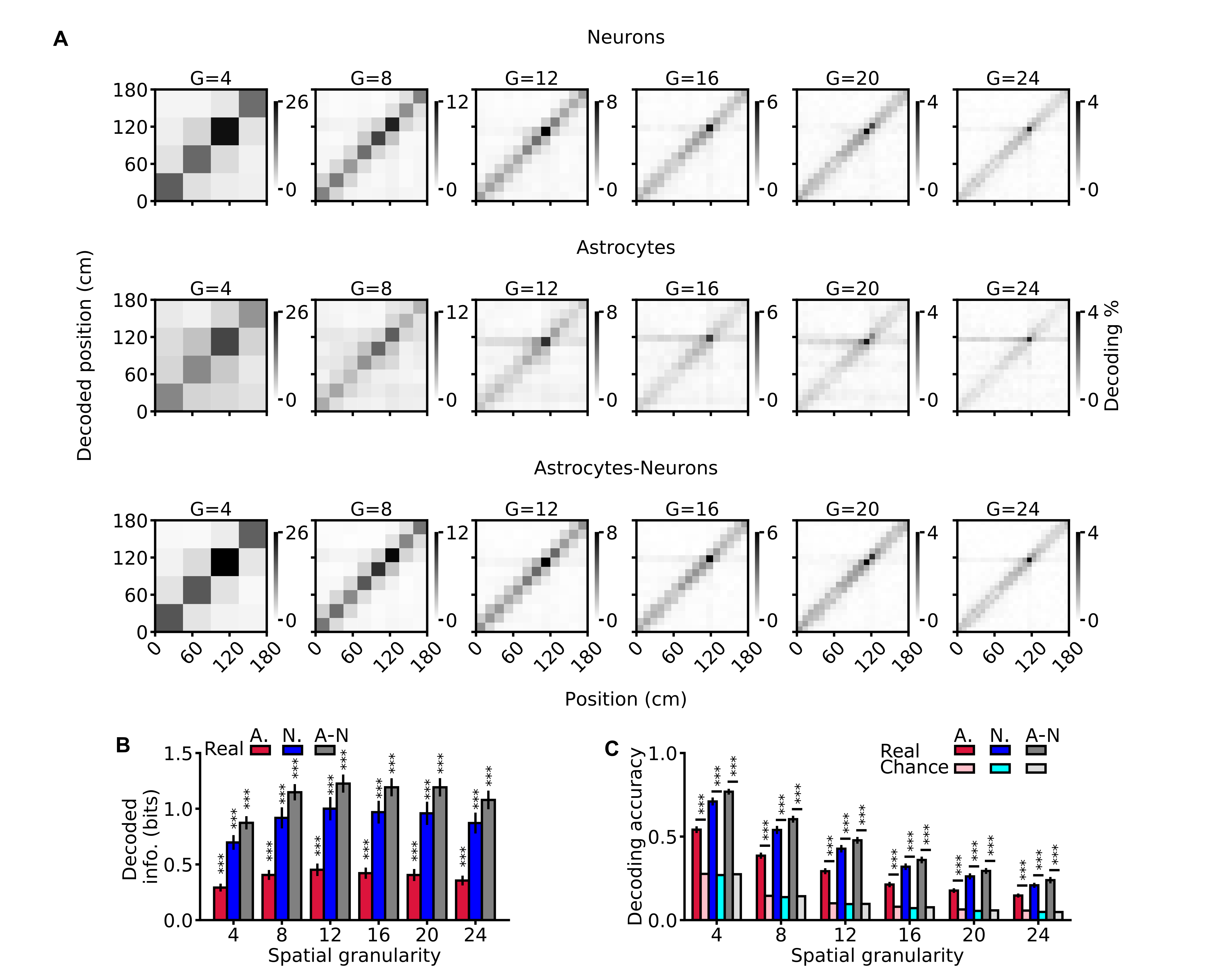

Supplement: S22 Fig — (A) Confusion matrices of an SVM classifier decoding the mouse’s position using population vectors comprising neuronal (top), astrocytic (middle), and neuronal + astrocytic ROIs (bottom) for various spatial granularities (G = 4, 8, 12, 16, 20, 24). The true position of the animal is shown on the x-axis and the decoded position on the y-axis. Gray scale indicates the percentage of occurrence of each matrix element. (B) Decoded mutual information between predicted and real position in the linear track and (C) decoding accuracy for the different population vectors as a function of spatial granularity. In B and C, asterisks indicate significance against chance level (S5 and S10 Tables). Data are displayed as mean ± SEM and were collected in 11 imaging sessions from 7 animals. The data presented in this figure can be found in S5 Data. ROI, region of interest; SEM, standard error of the mean; SVM, support vector machine. (TIFF) [file pbio.3001530.s022.tiff]
