# Supplementary material for: Complementary encoding of spatial information in hippocampal astrocytes
Source: PLoS Biol. 2022 Mar 3;20(3):e3001530. doi: 10.1371/journal.pbio.3001530 (PMC8893713; doi:10.1371/journal.pbio.3001530)
Supplement: S1 Table — (DOCX) [file pbio.3001530.s023.docx]

| Question | Approach | Results | Controls | Figures |
| --- | --- | --- | --- | --- |
| Do astrocytes encode spatial information in their intracellular Ca^2+^ dynamics? | - Combination of functional two-photon microscopy and virtual spatial navigation in head-fixed mice. - Test spatial information content and spatial tuning properties of CA1 astrocytic Ca^2+^ signals during monodirectional virtual navigation. - Test spatial information content and spatial tuning properties of CA1 astrocytic Ca^2+^ signals during bidirectional virtual navigation. | - In the mouse hippocampus, astrocytic Ca^2+^ signals encode information about position in space during virtual navigation. - Astrocytic spatial response profiles are reliable. - Astrocytic spatial response profiles tile the whole virtual corridor. - Astrocytic calcium dynamics convey information about position beyond proximal visual cues location. | - Quantification of information content is performed across a grid of 77 binning parameter combinations. - Non-parametric permutation testing is used to assess statistical significance. - Astrocytic spatial responses are robust to resampling approaches. - Non-parametric testing is used to assess statistical difference between decoding results before and after disruption of position within single visual cues. | Fig 1, 4.  S2 Fig, S5 Fig, S10 Fig. |
| Is spatial information encoded in astrocytic Ca^2+^ signals organized at the subcellular level? | - Classification of astrocytic subcellular ROIs according to their anatomical identity (somata vs. processes) to quantitatively compare spatial tuning properties at the subcellular level. | - Astrocytic somata and processes show significant spatial information encoding in their responses. - Single astrocytes can have different place fields in distinct topographically organized subcellular locations. | - Quantification of information content is performed across a grid of 77 binning parameter combinations. - Non-parametric permutation testing is used to assess statistical significance. | Fig 2.  S4 Fig, S6 Fig. |
| Can spatial information encoded in astrocytic Ca^2+^ signals be used to decode animals’ position? | - Deployment of a support vector machine (SVM) model to perform classification of animals’ position given a set of astrocytic Ca^2+^ signals: - during monodirectional virtual navigation. - during bidirectional virtual navigation. - Quantification of the impact of astrocytic Ca^2+^ signal correlations on the decoding of animals’ position. | - Animals’ position is efficiently decoded from astrocytic calcium signals. - Disruption of signal correlations of astrocytic population vectors reduce information content. | - Decoding analysis is performed across multiple classification granularities. - Decoding results are systematically above chance level estimates using non-parametric permutation testing for all granularities. - Non-parametric testing is used to assess statistical difference between decoding results before and after disruption of signal correlations. | Fig 3.  S8 Fig, S9 Fig.  S2 Table, S6 Table. |
| How does astrocytic representation of spatial information relate to that of neuronal cells? | - Combination of dual color functional two-photon microscopy and virtual spatial navigation in head fixed mice to simultaneously image astrocytic and neuronal activity. - Quantitative comparison of astrocytic and neuronal spatial tuning properties during monodirectional virtual navigation. | - Astrocytic and neuronal response profiles cover the virtual corridor - The majority of spatial information in astrocytes and neurons is genuine spatial information that cannot be explained by tuning to visual cues | - Quantification of information content is performed across a grid of 77 binning parameters combinations. - Non-parametric permutation testing is used to assess statistical significance. - Non-parametric testing is used to assess statistical difference between decoding results before and after disruption of position within single visual cues. | Fig 5.  S12 Fig, S14 Fig, S15 Fig, S17 Fig, S18 Fig.  S7 Table, S8 Table, S9 Table. |
| Is the information encoded in astrocytic calcium signals a redundant representation of space-encoding neuronal activity? | - Pairwise investigation of information encoding comparing astrocytic, neuronal, and mixed ROI pairs. - Information Breakdown analysis - Deployment of a support vector machine (SVM) model to perform classification of animals’ position given different sets of Ca^2+^ signals during monodirectional virtual navigation: - Using astrocytic signals. - Using neuronal signals. - Using both astrocytic and neuronal signals. - Quantification of the impact of Ca^2+^ signal correlations on position decoding. | - Astrocytic and neuronal responses encode information sharing, a position-dependent correlation component. - Astrocytic and neuronal spatial responses provide synergistic and complementary spatial information. - Astrocytes carry information about space that is not available in any of the nearby neurons. | - Decoding analysis is performed across multiple classification granularities. - Decoding results are systematically above chance level estimates using non-parametric permutation testing for all granularities. - Non-parametric testing is used to assess statistical difference between decoding results before and after disruption of signal correlations. | Fig 6.  S19 Fig, S20 Fig, S21 Fig, S22 Fig.  S3 Table, S4 Table, S5 Table, S7 Table, S10 Table. |
