# Supplementary material for: Complementary encoding of spatial information in hippocampal astrocytes
Source: PLoS Biol. 2022 Mar 3;20(3):e3001530. doi: 10.1371/journal.pbio.3001530 (PMC8893713; doi:10.1371/journal.pbio.3001530)
Supplement: S2 Table — p-values for one-tailed nonparametric permutation tests as a function of decoding granularity for decoded information (see Fig 3B) and decoding accuracy (S6 Fig). For each imaging session and each granularity, null distributions were obtained with 1,000 and 500 iterations to estimate chance level and trial shuffling, respectively (see Methods). Data from 7 imaging sessions from 3 animals. The data presented in this figure can be found in S1 Data. (DOCX) [file pbio.3001530.s024.docx]

|  | **Permutation type** | **p**  **G = 4** | **p**  **G = 8** | **p**  **G = 12** | **p**  **G = 16** | **p**  **G = 20** | **p**  **G = 24** |
| --- | --- | --- | --- | --- | --- | --- | --- |
| **Decoded**  **Information** | Chance | 1E-3 | 1E-3 | 1E-3 | 1E-3 | 1E-3 | 1E-3 |
|  | Trial-shuff. | 2E-3 | 2E-3 | 2E-3 | 2E-3 | 2E-3 | 2E-3 |
| **Decoding Accuracy** | Chance | 1E-3 | 1E-3 | 1E-3 | 1E-3 | 1E-3 | 1E-3 |
|  | Trial-shuff. | 2E-3 | 2E-3 | 2E-3 | 2E-3 | 2E-3 | 2E-3 |
