# Supplementary material for: Complementary encoding of spatial information in hippocampal astrocytes
Source: PLoS Biol. 2022 Mar 3;20(3):e3001530. doi: 10.1371/journal.pbio.3001530 (PMC8893713; doi:10.1371/journal.pbio.3001530)
Supplement: S3 Table — Information about position carried by pairs of ROIs (I) is compared to the sum (ILIN) or to the maximum (IMAX) of the information separately encoded by each member of the pair. A-A, pair composed of two astrocytic ROIs; N-N, pair composed of two neuronal ROIs; A-N, mixed pair composed of one astrocytic and one neuronal ROI. We summarize mean difference between groups, confidence interval limits, Cohen d effect size estimate, and p-value for Wilcoxon signed rank test. Information measures were corrected using two bias correction procedures, QE, and shuffled. Data are from 11 imaging sessions on 7 animals. The data for this table can be found in S1 Data and S5 Data. QE, quadratic extrapolation; ROI, region of interest. (DOCX) [file pbio.3001530.s025.docx]

| Pair type | Comparison | Mean | Lower-bound (bits) | Upper-bound (bits) | Cohen’s d | p | Bias correction procedure |
| --- | --- | --- | --- | --- | --- | --- | --- |
| A-A | I-I_LIN_ | 0.0037 | 0.0036 | 0.0038 | 1.847 | 1E-3 | QE |
| N-N | I-I_LIN_ | 0.0029 | 0.0028 | 0.0030 | 1.327 | 5E-3 | QE |
| A-N | I-I_LIN_ | 0.0052 | 0.0050 | 0.0053 | 2.032 | 1E-3 | QE |
| A-A | I-I_MAX_ | 0.0068 | 0.0065 | 0.0070 | 1.579 | 1E-3 | QE |
| N-N | I-I_MAX_ | 0.0182 | 0.0175 | 0.0185 | 2.280 | 1E-3 | QE |
| A-N | I-I_MAX_ | 0.0114 | 0.0111 | 0.0118 | 2.171 | 1E-3 | QE |
| A-A | I-I_LIN_ | 0.0016 | 0.0015 | 0.0017 | 1.013 | 1E-2 | Shuffled |
| N-N | I-I_LIN_ | 0.0025 | 0.0024 | 0.0027 | 1.133 | 7E-3 | Shuffled |
| A-N | I-I_LIN_ | 0.00233 | 0.00226 | 0.00240 | 1.723 | 1E-3 | Shuffled |
| A-A | I-I_MAX_ | 0.0046 | 0.0042 | 0.0047 | 1.083 | 5E-3 | Shuffled |
| N-N | I-I_MAX_ | 0.0174 | 0.0169 | 0.0179 | 2.099 | 1E-3 | Shuffled |
| A-N | I-I_MAX_ | 0.0087 | 0.0084 | 0.0090 | 2.006 | 1E-3 | Shuffled |
