# Supplementary material for: Complementary encoding of spatial information in hippocampal astrocytes
Source: PLoS Biol. 2022 Mar 3;20(3):e3001530. doi: 10.1371/journal.pbio.3001530 (PMC8893713; doi:10.1371/journal.pbio.3001530)
Supplement: S4 Table — p-values for two-tailed paired t tests with Bonferroni correction for decoded information of animal’s spatial location from population vectors comprising all astrocytic ROIs versus all ROIs of both types (top row) and all neuronal ROIs versus all ROIs of both types (bottom row) during monodirectional virtual navigation shown in Fig 6. Data from 11 imaging sessions from 7 animals. The data for this table can be found in S1 Data and S5 Data. ROI, region of interest. (DOCX) [file pbio.3001530.s026.docx]

|  | **p**  **G = 4** | **p**  **G = 8** | **p**  **G = 12** | **p**  **G = 16** | **p**  **G = 20** | **p**  **G = 24** |
| --- | --- | --- | --- | --- | --- | --- |
| **Astrocytes vs.**  **Astrocytes + Neurons** | 6E-7 | 6E-7 | 4E-7 | 6E-7 | 6E-7 | 1E-6 |
| **Neurons vs.**  **Astrocytes + Neurons** | 3E-4 | 5E-4 | 2E-3 | 1E-3 | 1E-3 | 2E-3 |
