# Supplementary material for: Complementary encoding of spatial information in hippocampal astrocytes
Source: PLoS Biol. 2022 Mar 3;20(3):e3001530. doi: 10.1371/journal.pbio.3001530 (PMC8893713; doi:10.1371/journal.pbio.3001530)
Supplement: S5 Table — p-values for one-tailed nonparametric permutation tests for decoding information from population vectors comprising either all astrocytic (top row), all neuronal (middle row), or ROIs of both types (bottom row) during monodirectional virtual navigation (see Fig 6 and S13 Fig). Significance levels are reported as a function of decoding granularity. For each imaging session and each granularity, null distributions were obtained with 1,000 and 500 iterations to estimate chance level and trial shuffling, respectively (Methods). Data from 11 imaging sessions from 7 animals. The data for this table can be found in S1 Data and S5 Data. ROI, region of interest. (DOCX) [file pbio.3001530.s027.docx]

|  | **Permutation type** | **p**  **G = 4** | **p**  **G = 8** | **p**  **G = 12** | **p**  **G = 16** | **p**  **G = 20** | **p**  **G = 24** |
| --- | --- | --- | --- | --- | --- | --- | --- |
| **Astrocytes**  **(A)** | Chance | 1E-3 | 1E-3 | 1E-3 | 1E-3 | 1E-3 | 1E-3 |
|  | Trial-shuff. | 2E-3 | 2E-3 | 2E-3 | 2E-3 | 2E-3 | 2E-3 |
| **Neurons**  **(N)** | Chance | 1E-3 | 1E-3 | 1E-3 | 1E-3 | 1E-3 | 1E-3 |
|  | Trial-shuff. | 2E-3 | 2E-3 | 2E-3 | 2E-3 | 2E-3 | 2E-3 |
| **Astrocytes**  **+**  **Neurons (A-N)** | Chance | 1E-3 | 1E-3 | 1E-3 | 1E-3 | 1E-3 | 1E-3 |
|  | Trial-shuff. | 2E-3 | 2E-3 | 2E-3 | 2E-3 | 2E-3 | 2E-3 |
