# Supplementary material for: Complementary encoding of spatial information in hippocampal astrocytes
Source: PLoS Biol. 2022 Mar 3;20(3):e3001530. doi: 10.1371/journal.pbio.3001530 (PMC8893713; doi:10.1371/journal.pbio.3001530)
Supplement: S6 Table — p-values for one-tailed nonparametric permutation tests as a function of decoding granularity for decoded information (see S7B and S7F Fig) and decoding accuracy (see S7C and S7G Fig). Decoding performance is reported for forward- and backward-running directions (see S7 Fig). For each imaging session and each granularity, null distributions were obtained with 1,000 iterations to estimate chance level (Methods). Data from 15 imaging sessions in 4 animals for forward-running direction. Data from 17 imaging sessions in 4 animals for backward-running direction. The data for this table can be found in S3 Data. (DOCX) [file pbio.3001530.s028.docx]

|  | **Direction** | **p**  **G = 4** | **p**  **G = 8** | **p**  **G = 12** | **p**  **G = 16** |
| --- | --- | --- | --- | --- | --- |
| **Decoded information** | Forward | 1E-3 | 1E-3 | 1E-3 | 1E-3 |
|  | Backward | 1E-3 | 1E-3 | 1E-3 | 1E-3 |
| **Decoding accuracy** | Forward | 1E-3 | 1E-3 | 1E-3 | 1E-3 |
|  | Backward | 1E-3 | 1E-3 | 1E-3 | 1E-3 |
