# Supplementary material for: Complementary encoding of spatial information in hippocampal astrocytes
Source: PLoS Biol. 2022 Mar 3;20(3):e3001530. doi: 10.1371/journal.pbio.3001530 (PMC8893713; doi:10.1371/journal.pbio.3001530)
Supplement: S7 Table — Descriptive statistics and confidence intervals estimation for pairwise Pearson correlation. Mean, SEM, 95% confidence interval limits, and p-value for Wilcoxon rank sums test for H0 = 0 are shown. Pairs were composed either of 2 astrocytic ROIs belonging to the same astrocyte (A-Asame), 2 astrocytic ROIs belonging to the different astrocytes (A-Aother), 2 neuronal ROIs (N-N), or one astrocytic and one neuronal ROI (A-N). Correlation was measured for ROI pairs with reliable spatial information or for all possible pairs. Data are from 11 imaging sessions on 7 animals. The data for this table can be found in S4 Data. ROI, region of interest; SEM, standard error of the mean. (DOCX) [file pbio.3001530.s029.docx]

| **Pair type** | **Class** | **Mean**  **pairwise correlation** | **s.e.m.** | **Lower-bound** | **Upper-bound** | **p** |
| --- | --- | --- | --- | --- | --- | --- |
| **Astrocytic ROIs from same cell (A-A_same_)** | Reliably encoding spatial information | 0.68 | 0.06 | 0.57 | 0.78 | 2E-4 |
| **Astrocytic ROIs from different cells (A-A_other_)** | Reliably encoding spatial information | 0.31 | 0.06 | 0.20 | 0.42 | 3E-3 |
| **Neurons**  **(N)** | Reliably encoding spatial information | 0.10 | 0.01 | 0.08 | 0.12 | 7E-5 |
| **Astrocytes**  **+**  **Neurons**  **(A-N)** | Reliably encoding spatial information | 0.05 | 0.02 | 0.02 | 0.08 | 3E-3 |
| **Astrocytic ROIs from same cell (A-A_same_)** | All | 0.52 | 0.03 | 0.47 | 0.57 | 7E-5 |
| **Astrocytic ROIs from different cells (A-A_other_)** | All | 0.28 | 0.03 | 0.22 | 0.34 | 2E-4 |
| **Neurons**  **(N)** | All | 0.07 | 0.01 | 0.05 | 0.09 | 7E-5 |
| **Astrocytes**  **+**  **Neurons**  **(A-N)** | All | 0.04 | 0.01 | 0.01 | 0.06 | 1E-3 |
