# Supplementary material for: Complementary encoding of spatial information in hippocampal astrocytes
Source: PLoS Biol. 2022 Mar 3;20(3):e3001530. doi: 10.1371/journal.pbio.3001530 (PMC8893713; doi:10.1371/journal.pbio.3001530)
Supplement: S8 Table — p-values for binomial tests for astrocytic (top row) or neuronal (bottom row) ROIs encoding reliable spatial information showing a significant decrease in their information content when position was shuffled within individual visual cues (see also S14 Fig). Significance levels are reported as a function of the number of position bins (NS). For each imaging session and each NS, IV distributions were obtained with 100 iterations in which position was shuffled within visual cues to estimate average IV (see also Methods). Data from 11 imaging sessions from 7 animals. The data for this table can be found in S5 Data. ROI, region of interest. (DOCX) [file pbio.3001530.s030.docx]

|  | **p**  N_S_ **= 9** | **p**  N_S_ **= 12** | **p**  N_S_ **= 15** | **p**  N_S_ **= 18** |
| --- | --- | --- | --- | --- |
| **Astrocytes**  **(A)** | 2E-17 | 2E-13 | 1E-11 | 2E-14 |
| **Neurons**  **(N)** | 5E-270 | 4E-259 | 5E-245 | 3E-243 |
