# Supplementary material for: Complementary encoding of spatial information in hippocampal astrocytes
Source: PLoS Biol. 2022 Mar 3;20(3):e3001530. doi: 10.1371/journal.pbio.3001530 (PMC8893713; doi:10.1371/journal.pbio.3001530)
Supplement: S9 Table — p-values for Wilcoxon signed rank tests for decoding information from population vectors comprising either all astrocytic (top row) or all neuronal (bottom row) ROIs during monodirectional virtual navigation (see S21 Fig). Significance levels are reported as a function of decoding granularity. For each imaging session and each granularity, IV distributions were obtained with 500 iterations in which position was shuffled within visual cues to estimate average IV (see Methods). Data from 11 imaging sessions from 7 animals. The data for this table can be found in S5 Data. ROI, region of interest. (DOCX) [file pbio.3001530.s031.docx]

|  | **Permutation type** | **p**  **G = 9** | **p**  **G = 12** | **p**  **G = 15** | **p**  **G = 18** |
| --- | --- | --- | --- | --- | --- |
| **Astrocytes**  **(A)** | Position within visual cues | 9.8E-04 | 9.8E-04 | 9.8E-04 | 9.8E-04 |
| **Neurons**  **(N)** | Position within visual cues | 9.8E-04 | 9.8E-04 | 9.8E-04 | 9.8E-04 |
