# Supplementary material for: Complementary encoding of spatial information in hippocampal astrocytes
Source: PLoS Biol. 2022 Mar 3;20(3):e3001530. doi: 10.1371/journal.pbio.3001530 (PMC8893713; doi:10.1371/journal.pbio.3001530)
Supplement: S10 Table — p-values for one-tailed nonparametric permutation tests for decoding accuracy from population vectors comprising either all astrocytic ROIs (top row), all neuronal ROIs (middle row), or all ROIs of both types (bottom row) during monodirectional virtual navigation (see S13 Fig). Significance levels are reported as a function of decoding granularity. For each imaging session and each granularity, null distributions were obtained with 1,000 iterations to estimate chance level (Methods). Data from 11 imaging sessions from 7 animals. The data for this table can be found in S1 Data and S5 Data. ROI, region of interest. (DOCX) [file pbio.3001530.s032.docx]

|  | **Permutation type** | **p**  **G = 4** | **p**  **G = 8** | **p**  **G = 12** | **p**  **G = 16** | **p**  **G = 20** | **p**  **G = 24** |
| --- | --- | --- | --- | --- | --- | --- | --- |
| **Astrocytes**  **(A)** | Chance | 1E-3 | 1E-3 | 1E-3 | 1E-3 | 1E-3 | 1E-3 |
| **Neurons**  **(N)** | Chance | 1E-3 | 1E-3 | 1E-3 | 1E-3 | 1E-3 | 1E-3 |
| **Astrocytes**  **+**  **Neurons**  **(A-N)** | Chance | 1E-3 | 1E-3 | 1E-3 | 1E-3 | 1E-3 | 1E-3 |
