# Supplementary material for: Complementary encoding of spatial information in hippocampal astrocytes
Source: PLoS Biol. 2022 Mar 3;20(3):e3001530. doi: 10.1371/journal.pbio.3001530 (PMC8893713; doi:10.1371/journal.pbio.3001530)
Supplement: Attachment — Submitted filename: Point-by-point response Curreli et al.docx [file pbio.3001530.s038.docx]

**Point-by-point response to the referees’ comments**

We thank the Reviewers for their positive and constructive feedback on our manuscript. We performed additional experiments and analyses to address all of the Referees’ comments. Based on the additional work suggested by the referees, we believe that we have improved our manuscript and significantly strengthened our findings. Please find below our point-by-point to their comments. The original Reviewer’s comments are shown in black. Author responses is shown in blue. Edits to the original manuscript are shown in red in the revised text.

***Reviewer #1***

*Reviewers’ comment: In this manuscript, authors have investigated the calcium activity of hippocampal astrocytes and neurons relate to the spatial information of mice navigating a virtual. To investigate this, authors have used genetically-encoded calcium indicators and simultaneous two-photon calcium imaging of hippocampal astrocytes and neurons in awake mice.*

*Authors developed and used a meticulous and complex analysis of the calcium activity of astrocyte somas and processes as well as neurons in relation to the virtual spatial location of the animal. The study provides an exhaustive characterization of the signals, which can be summarized in two important messages: 1) astrocyte activity in the somas and processes is modulated by the spatial position of the mice, indicating that they encode spatial information; 2) astrocytes have broader response field width and a different distribution of field position that neurons, suggesting a complementary and synergistic spatial information processed by astrocytes and neurons.*

*This is an exciting and elegant study that adds valuable information regarding a relevant topic in current neuroscience, the physiology of astrocytes and their active involvement in brain function. More specifically, the present demonstration that astrocytes encode spatial information, i.e., astrocytes act like place cells, is a break-through finding that change our view of information processing in the brain.*

*The manuscript presents a very exhaustive and deep study that presents novel and interesting results, which are very convincing. Furthermore, the study is methodologically and technically adequate, with elegant and appropriate experimental design to provide robust results. The conclusions reached are well-supported by the experimental data.*

*Therefore, I have few comments about present manuscript. While it presents many strengths, I have also found some weaknesses that need to be addressed to improve the already high quality of the manuscript.*

*In summary, the results presented are novel and relevant. I believe that present manuscript will positively and strongly impact the neuroscience field. It has high merit to be published in PLOS Biology.*

Author response: we thank the Referee for the positive evaluation of our work.

*Specific comments*

*Reviewers’ comment: 1. When analyzing calcium events, authors describe in Methods: "for each trace, the standard deviation (σ1) of the signal was computed and values crossing threshold set at ± σ1 were removed from the trace. This procedure excluded large transients". Authors should provide rationale about why these large events were excluded. If they are not excluded, would the results presented still be valid?*

Author response: we apologize with the Referee for not being sufficiently clear in our previous version of the text. Large events were excluded only for the computation of the standard deviation (σ_2_) to avoid large biases in its estimation. Once σ_2_ was computed, the analysis was performed on the whole event trace (including all events). We modified the text to make this clear and to better explain the rationale behind our choice. The edited text reads (lines 618-627):

“For both astrocytic and neuronal fluorescence traces, extraction of statistically significant calcium events was performed on ΔF/F_0_ traces via modified implementation of the algorithm described in (25). In brief, for each trace, a first parameter (σ_1_) was computed as the standard deviation of the whole signal. Values crossing the threshold set at ± σ1 were removed from the trace and a second parameter (σ_2_) was computed as the standard deviation of the thresholded trace. This procedure avoided biases induced by large signal transients on the estimation of the signal standard deviation in the absence of transients and provided a better estimation of signal baseline fluctuations (σ_2_). For astrocytic traces, fluorescence transients were identified on the original trace (thus considering all data) as events that: *i)* crossed the threshold of ± 2σ_2_; *ii)* returned within ± σ_2_ in more than 0.5 s (25).”

*Reviewers’ comment: 2. To simultaneously monitor astrocyte and neuronal activity, authors delivered virus containing different genetically encoded calcium indicators under cell-specific promoters that are expected to provide cell-specific expression. Although this approach is widely and confidently used, authors should provide immunohistochemistry quantification that this is the case in their own hands. This is a very simple control that is needed to support the validity of the data.*

Author response: we thank the referee for raising this point. We performed immunohistochemical analysis to confirm specificity of the constructs we used. Specifically:

a) In mice injected with pZac2.1 gfaABC1D-cyto-GCaMP6f, we stained sections of fixed tissue with an anti-GFAP antibody and a red-shifted secondary antibody. We found that almost all GCaMP6f-expressing cells were positive for GFAP (95 ± 7 %, out of a total of 45 GCaMP6f-expressing cells from N = 6 sections from 3 mice). This result confirms the astrocyte specificity of the pZac2.1 gfaABC1D-cyto-GCaMP6f construct and it was added to the manuscript (new extended data Fig. 1).
b) In mice injected with pZac2.1 gfaABC1D-cyto-GCaMP6f and pAAV-CAMKII-jRCaMP1a, we stained sections of fixed tissue with either an anti-NeuN antibody and an infrared shifted secondary antibody, or an anti-GFAP antibody and an infrared shifted secondary antibody. We found that the large majority of jRCaMP1a expressing cells were positive for NeuN (93 ± 8 %, out of a total of 985 jRCaMP1a-expressing cells from N = 6 sections from 3 mice), confirming neuronal expression of the red-shifted indicator. We also found that virtually all GCaMP6f-expressing cells were positive for GFAP (100 ± 0 %, out of a total of 71 GCaMP6f-expressing cells, N = 6 sections from 3 mice). Importantly, we did not detect GCaMP6f-expressing cells, which were stained for NeuN (0 ± 0 %, 0 out of a total of 50 GCaMP6f-expressing cells, from N = 6 sections from 3 mice), confirming non-neuronal expression of GCaMP6f (see point a) above). These findings are displayed in new extended data Fig. 11.

A paragraph (lines 954-956) to describe the methods used in these measurements.

*Reviewers’ comment: 3. The analysis used is certainly complex and represents a tour de force, probably necessary to reveal the results obtained. It is perfectly defined and provide robust results. Yet, one could wonder whether such results may be an "artifact" of the analysis. Authors could use some controls to discard this possibility. For example, how is the astrocyte and neuronal activities in a static position with absence of virtual navigation? This is probably an unnecessary control, but it may enhance the robustness of the results by discarding some potential analytical artifacts.*

Author response: we thank the referee for raising this point and for encouraging us to include this important control experiment. In two new figures (extended data Fig. 4 and extended data Fig. 13), we now show the amplitude of astrocytic and neuronal calcium activities when the mouse was still vs. when the mouse was locomoting (both in the presence of the virtual reality, extended data Fig. 4A and extended data Fig. 13A) and in the absence vs. presence of virtual reality (extended data Fig. 4B and extended data Fig. 13B). We found that astrocytic calcium events were smaller when the mouse was still vs. when the mouse was locomoting and in the absence vs. presence of virtual reality. This was observed both in mice expressing only GCaMP6 into astrocytes (extended data Fig. 4A, B) and in mice expressing GCaMP6 into astrocytes and jRCaMP1a into neurons (Extended data Fig. 13 A, B). Taken together, these results (described at lines 89-91 and 194-196) show that the events that we recorded when the mouse was engaged in virtual navigation are larger than those spontaneously occurring.

*Reviewers’ comment: 4. In several instances, authors seem to imply that astrocytic encoding of spatial information is involved in the brain information processing. For example, in page 4, line 78: "Thus, astrocytic calcium signaling participates in encoding spatial information in the hippocampus". While I am convinced by the results that astrocytes encode spatial information, the fact that this is translated to the information processing of the hippocampus has not been actually tested. This is an interesting hypothesis, but it would require additional studies, certainly out of the scope of the present manuscript, such as the specific manipulation of the astrocyte activity and analysis of the outcome. Unless authors perform such studies, they should town down the interpretation of the results in the Results section, restricting such hypothetical ideas to the Discussion.*

Author response: We carefully checked the Results section, and we found no claim of astrocytes being involved in the processing of brain information. All speculations and considerations about the implications of our results for brain information processing are confined to the discussion and explicitly presented as hypotheses that remain to be proved by further work. The sentence indicated by the referee ("Thus, astrocytic calcium signaling participates in encoding spatial information in the hippocampus") was written to imply that astrocyte encode information about the external space, but not that they participate in processing of information and in information transfer to neurons. To make double sure that our sentence could not be misinterpreted, we revise it to the simpler and more conservative “Thus, hippocampal astrocytic calcium signaling encoded spatial information” (line 105).

*Minor comments*

*Reviewers’ comment: 1. The wording of the title may be confusing. "Glial place cells" is not completely proper. There are several types of glial cells, but the study is focused on astrocytes.*

Author response: the current title “Glial place cells: complementary encoding of spatial information in hippocampal astrocytes” does specify that the glial cell type we describe in this study are astrocytes. We believe mentioning glia at the beginning of the title is important to capture the attention of the neural place cell community and we would thus prefer to keep the title as is.

***Reviewer #2***

*Reviewers’ comment: This paper shows for the first time that astrocytes in the CA1 region of the hippocampus have place fields that tile a virtual environment in similar ways to neuronal place cells. Further, they show that astrocytic processes from the same astrocytes can have differences in their place field locations relative to the cell body. Lastly, they claim that astrocytes carry unique spatial information from neuronal populations that, synergistically with neurons, enhances the overall spatial encoding properties of the CA1 region. This is new information that makes a significant contribution to the field and opens new avenues to explore as astrocytes have been left out of most theories of hippocampal function as we did not know their encoding properties. However, some of their conclusions are supported by data that I believe needs further analysis to be convincing.*

*These comments are in the order in which I read the manuscript, not in order of significance:*

Author response: we thank the Referee for their positive evaluation of our work.

*Reviewers’ comment:*

** They show mean astrocyte activity as a function of position (Fig, 1E), but it would be informative to see trial-to-trial traces to get an idea of their variability considering this is the first time the field is seeing astrocytic place fields (PFs).*

Author response: following the Referee’s comment, we now provide examples of multiple traces to display trial-to-trial variability in astrocytic place fields (new extended data Fig. 3A).

*Reviewers’ comment:*

** Along these lines, they should quantify trial-to-trial parameters of these astrocyte PFs such as overdispersion1 and precision2 and compare them to neuronal PFs.*

Author response: we thank the Referee for this suggestion. We now compute spatial precision as in (Sheffield et al. 2015) and include these results in the manuscript (new extended data Fig. 3D). We also evaluated overdispersion (Fenton et al. 2010), but decided not to use it because this parameter heavily relies on the statistical properties of spike discharges that are fundamentally different for calcium events. Using spatial precision, we found (new extended data Fig. 3D) that calcium responses in astrocytic ROIs encoding reliable spatial information were moderately more precise than their unmodulated counterpart (median ± m.a.d. 3.2E-2 ± 0.6E-2, N = 155 out of 356 total ROIs, for ROIs with reliable spatial information, black; 3.0E-2 ± 0.5E-2 cm^-1^, N = 201 out of 356 total ROIs, for not-modulated ROIs, grey: p = 3.8E-2, Kolmogorov-Smirnov test; 7 imaging sessions on 3 animals). These results are now presented at lines 79-84 of the main text.

As suggested by the referee, we also compared the spatial precision of astrocytic responses with that of neuronal responses. We found that the responses of position-encoding neurons were more precise than the responses of simultaneously recorded position-encoding astrocytic ROIs (mean ± s.e.m.; neuronal responses 7.5E-2 ± 1.6E-2; astrocytic responses 4.1E-2 ± 0.2E-2; p = 4.6E-2 Wilcoxon signed-rank test; 11 imaging sessions on 7 animals). These data are included in new extended data Fig. 16A and are presented at lines 206-211. The Methods section has been updated at lines 723-742 to describe this new analysis.

*Reviewers’ comment:*

** And what about PF stability? Do they change, shift, or remap throughout a session? Neuronal PFs have been shown to shift in CA1 and even remap over longer time periods. It would be good to analyze astrocytic PF dynamics in this way. They could be more or less stable than neuronal PFs, and that would be important to know.*

Author response: following the Referee’s comment, we investigated the stability of spatial responses within the timespan of our recording sessions (approximately one hour). We computed response fields using running trials recorded either during the first or the second half of each experimental session. As in (Sheffield et al. 2015), we considered as stable those response fields showing an absolute difference in the estimated response field centers < 15 cm. We found that a fraction of astrocytic ROIs (10 ± 10 %, 35 out of 356 ROIs, from 7 sessions in 3 animals) encoded reliable spatial information and had stable response field. We now provide this information in the main text (lines 87-88).

We also compared astrocytic response field stability with neuronal place field stability in simultaneous recordings from both cell types. We found that similar fractions of astrocytic ROIs and neuronal cells encoded reliable spatial information and had stable response field (astrocytes, 8 ± 7 %, 29 out of 341 ROIs; neurons, 16 ± 9 %, 139 out of 870 ROIs; p = 0.29, Wilcoxon Rank-sum test; from 11 imaging sessions on 7 animals). We now include this information in extended data Fig 16B and describe it in the main text (lines 211-215).

*Reviewers’ comment:*

** A recent paper on bioRxiv3 shows CA1 astrocytes have a ramping to reward signal in an almost identical task as shown here. Do the authors also see these signals? In addition, is there an over-representation of the reward zone in astrocytic PFs similar to what is seen with neuronal PFs?*

Author response: thanks for this comment. In Doron et al. BioRxiv 2021, the authors reported two main findings. First, in expert mice engaged in a virtual reality navigation task they observed a ramping increase in the number of astrocytic ROIs showing simultaneously occurring calcium events (named by the authors “concurrent events”) towards the reward location. Second, they reported that a fraction of ROIs showed significant information about position. To address the point raised by the Referee, we then aimed to identify ROIs characterized by a “ramping signal” towards the reward zone (as shown in Fig. 2E of Doron et al. BioRxiv 2021). It must be noted that precisely replicating the analytical approach described in Doron et al. BioRxiv 2021 was difficult because of the succinct description of analytical methods in the preprint. On ROIs that we previously identified as being reliably encoding spatial information, we binarized calcium event traces, setting to 1 signal values belonging to a calcium event and to 0 otherwise. We used the binarized event trace to compute response profiles (as previously done in our study, see Methods). We set the spatial bin width to 18 cm, a value similar to that (17 cm) reported by Doron et al. BioRxiv 2021. We found (Fig. R1-R2) that a subset of ROIs encoding reliable spatial information were characterized by event probability maximizing close to the position of reward delivery (5 ± 8 %; N = 19 out of 356 ROIs, 7 imaging sessions from 3 animals). Thus, this new preliminary analysis suggest that we observed a phenomenon similar to the one reported in Doron et al. BioRxiv 2021 in a subpopulation of astrocytic ROIs. It must be noted, however, that whether this fraction of ROIs genuinely reported reward encoding signals is debatable, because in our data it is not possible to decouple reward information from spatial information (an argument that we believe applies also to the results in Doron et al. BioRixv 2021). For the reason described above, we present these results for the referee’s inspection only.

**Figure R1. Astrocytic event probability during virtual navigation.** (A) Normalized probability of calcium event as a function of position for astrocytic ROIs with reliable spatial information (n = 155 ROIs with reliable spatial information out of 356 total ROIs, from 7 imaging sessions in 3 animals). ROIs are ordered according to the position of the maximum of the normalized event probability function. Red dots highlight ROIs encoding reliable spatial information showing a ramping signal towards the reward location, dotted lines highlight reward position (115 cm; yellow) and reward area limits (lower bound = 90 cm, upper bound = 144 cm; magenta). Vertical scale bar: 50 ROIs.

**Figure R2. Astrocytic event probability during virtual navigation in “ramping ROIs”.** Probability of calcium event as a function of position for ”ramping” astrocytic ROIs with reliable spatial information (n = 19 ROIs with reliable spatial information and ramping response out of 356 total ROIs, from 7 imaging sessions in 3 animals). Dotted lines highlight reward position (115 cm; yellow) and reward area limits (lower bound = 90 cm, upper bound = 144 cm; magenta). The green dots indicate the maximum of the event probability function.

*Reviewers’ comment:*

** What is their interpretation of what underlies astrocytic calcium signals? What is the source of calcium? What membrane potentials are likely leading to the calcium signals they see? What is thought to drive depolarizations in astrocytes and how do they differe to the inputs driving CA1 pyramidal cells? A discussion on how the authors think about these signals is needed.*

Author response: we thank the Referee for raising these important questions, which have been the focus of intense research over the last 30 years. We now added a paragraph discussing the molecular and cellular mechanisms that may underlie the generation of the astrocytic calcium dynamics that we describe in the manuscript. The new text reads (lines 283-308):

“Since the seminal observations of Cornell-Bell et al. (37) and Charles et al. (38), it has been shown that astrocytic calcium signaling can be activated by the extracellular increase in the concentration of neuroactive molecules (1, 39, 40). The source of the observed calcium signaling has long been investigated and there is evidence of both release from intracellular organelles (e.g., endoplasmic reticulum and mitochondria, (6, 41) and direct calcium influx through the astrocytic plasma membrane (42). Moreover, while neuronal activity induces membrane depolarization in astrocytes, these depolarizations are small (a few millivolts in maximal amplitude), at least at the level of the astrocytic somata (2, 43, 44). Pharmacological studies demonstrated that these membrane potential depolarizations are mediated mostly by K^+^ conductances and transporters-mediated currents (45). For neuronal activity-induced calcium signals, a largely accepted model (39, 40) is thus that spillover of neurotransmitter release at the synaptic cleft activates receptors in the plasma membrane of astrocytic processes, which enwrap the pre- and the post-synaptic terminals. Activation of astrocytic receptors then causes the mobilization of intracellular calcium signaling. Within this framework, it is interesting to note that previous studies in vivo showed that calcium dynamics into astrocytes largely mirror the activity of nearby neurons (7, 10, 11). In the present study, we instead show that spatial information encoded in astrocytes is complementary to that encoded by nearby neurons. This observation raises a series of questions about the molecular, anatomical, and network mechanisms that may generate the observed information complementarity. Can complementarity be generated by differences in the diffusion of pre-synaptically released neurotransmitter that reaches postsynaptic neuronal terminals in the synaptic cleft vs. thin astrocytic processes outside the cleft? Are the different molecular mechanisms regulating intracellular signaling in neurons and astrocytes accountable for the observed information complementarity? Additionally, can information complementarity stem from astrocytes integrating spatial information from different sets (or different combinations) of pre-synaptic terminals compared to post-synaptic neurons? Future studies combining experimental and modeling work will be fundamental to tackle these questions.

*Reviewers’ comment:*

** I think it is hard to interpret comparisons between calcium dynamics in Astrocytes and neurons (Fig. 4) as one is in due to underlying action potentials and the other is due to graded changes in Vm. Calcium buffering is also likely very different in astrocytes versus neurons (which they do briefly mention in the discussion). Also, GCaMP (astrocytes) and RGeCO (neurons) have different kinetics. Therefore, concluding that PF widths or any other properties are different is difficult and needs further experimental evidence (ephys). I am not asking them to do these experiments as they are extremely difficult, but I am asking them to temper their conclusions based on these confounds or do more analysis to better support their claims here.*

Author response: we agree with the referee and we removed our conclusion on the difference in place field properties between neurons and astrocytes at lines 138-156 and 222-229 in the Results section. The edited text now reads:

Lines 190-206

“For both astrocytes and neurons, the distribution of field position covered the entire length of the virtual corridor (Fig. 5C, D). The median width of the astrocytic spatial field was statistically larger than that of neurons (Fig. 5E, Extended data Table 1). Event triggered averages of astrocytic ROIs signals triggered by neuronal signals are shown in Extended data Fig. 12. Both, neuronal and astrocytic calcium events were bigger when the mouse was engaged in locomotion (Extended data Fig. 13A) and, for spatially modulated ROIs, in the presence vs. the absence of virtual reality (Extended data Fig. 13B). We then investigated the organization of astrocytic and neuronal spatial representations across the FOV. We found that calcium dynamics among mixed pairs of ROIs (one astrocytic ROI with reliable spatial information and one neuronal ROI with reliable spatial information) were significantly correlated (Extended data Fig. 14), independent of pair distance (0-160 µm; Fig. 5F). Correlation among pairs of astrocytic ROIs was generally higher than correlation among pairs of neuronal ROIs (Extended data Fig. 14, 15), even when we stratified the calculation of pairwise correlation for pairs of ROIs belonging to the same astrocyte and for pairs of ROIs belonging to different astrocytes (Extended data Fig. 14). The difference in spatial field position of an astrocytic ROI with reliable spatial information and a neuronal ROI with reliable spatial information was also largely independent of pair distance (Fig. 5G).”

Lines 224-226

“We then quantitatively tested whether calcium dynamics in astrocytes and neurons carry the same or complementary information about space. We did so at the pairwise level using mutual information analysis (27) on all pairs of ROIs (either astrocytic, neuronal, or mixed pairs).“

Lines 274-279

“Most importantly, combining astrocytic and neuronal signals generated significantly greater information about the animal’s position, indicating the signals are both complementary and synergistic. The complementary and synergistic information of astrocytes relied both on the diversity of position tuning and on position-dependent correlations among astrocytic and neuronal ROIs similarly to what observed on neuronal ROIs by (29).”

*Reviewers’ comment:*

** Supp Fig 9 shows the mean correlation of A-N pairs of cells with high spatial info, with the conclusion that they are significantly correlated above 0 (line 144). However, the effect size is very small. It would be much better to quantify this effect size using different analysis. I recommend the authors conduct estimation approaches on these datasets4. This emphasizes reporting effect sizes with expressions of uncertainty (interval estimates) to make better inferences: it pushes back against over-confident claims from inadequate samples, improves comparisons of results across contexts, and normalizes the publication of negligible effects4.*

Author response: following the Reviewer’s comment, we performed estimate approaches following (Calin-Jageman and Cumming, eNeuro 2019). The results of estimation approaches fully confirmed our previous results and can be found in Supplementary Tables 3 and 7, where we report mean and s.d. (or s.e.m.) with relative confidence intervals. The Methods section has also been updated at lines 976-978.

*Reviewers’ comment:*

** Lines 145-146 states "Correlation among pairs of astrocytic ROIs was generally higher than correlation among pairs of neuronal ROIs". But isn't this to be expected given the population of astrocytic ROIs comprises processes that are part of the same astrocyte? And even though the authors have found processes from the same astrocyte can have different PFs, they are still likely to be more correlated that PFs from other astrocytes. It is therefore not a fair comparison between these neuronal and astrocyte populations.*

Author response: we agree with the Referee’s comment. To address it, we broke down the calculation of pairwise correlation for all pairs of astrocytic ROIs into the calculation of pairwise correlation for pairs of ROIs belonging to the same astrocyte and into the calculation of pairwise correlation among ROIs belonging to different astrocytes (extended data Fig. 14 and Table 7). In both cases, we confirmed that correlation of pairs of astrocytic ROIs were higher than that of pairs of neuronal ROIs.

*Reviewers’ comment:*

** Fig 5A, again, the effect sizes for comparisons are very small in some cases. Basing conclusions on these is problematic. Estimation approaches should again be used here. This may influence a major conclusion of the paper which is stated in Line 160: "information carried by the pairs was also synergistic". With such small effect sizes the authors need to be very careful about making such a conclusion. Doing the estimation analysis will help in this regard and show how big or small these differences are, and conclusions should be based on that.*

Author response: we followed the Referee’s suggestion. We now provide the results of the analysis of pairwise information encoding pairing appropriate statistical tests with estimation approaches of difference and effect sizes. For each pair type, we report (new Table 3) the mean difference in information, the lower- and upper-bound confidence intervals computed by estimation, the Cohen’s d coefficient to quantify of effect size, and the results of the Wilcoxon signed-rank tests. These values are reported for both the I vs. I_LIN_ comparison and the I vs. I_MAX_ comparison. Overall, statistical testing, estimation approaches, and effect sizes are consistent one with the others, confirming our conclusion that spatial information encoded in astrocytic and neuronal networks is complementary and synergistic. The Methods section has been modified to describe this analysis at lines 976-978.

*Reviewers’ comment:*

** Lines 178-180 "Complementary and synergistic spatial information encoding in mixed pairs suggests that the astrocytic network carries additional information unique from that encoded in neuronal circuits also at the whole population level." But is this necessarily true? Does it really mean the astrocytic information is "unique" or is the information the same, it's just adding more of it to help the decoder? The authors should explain their thinking a little more on this point. Astrocytes are encoding place, so in that sense they are not unique. And decoder performance is always a function of the number of cells used. For instance, if they recorded from more place cells their decoder would perform better. Lines 187-188 states "This result proved that the population of astrocytic ROIs carries information not found in neurons or their interactions." But the authors didn't record from all neurons. And again, if they added more neurons their decoder would perform better. Would they then conclude that the extra neurons they recorded from were unique?*

Author response: we thank the Referee for raising these points.

The population decoding and information theoretic methods that we used, and that one of the authors (S.P.) has contributed to pioneer (with others) over the years, can indeed allow us to conclude that the astrocytes carry spatial information that was not found in the activity of neurons and their interactions. However, the referee is correct in pointing out that, because we did not record all the cells (astrocytes and neurons), we can only reach this kind of conclusion with regard to the cells that we imaged. To address this concern, we rephrased the sentence highlighted by the referee more conservatively and precisely, as follows (lines 248-250):

“Complementary and synergistic spatial information encoding in mixed pairs suggests that the network of astrocytes that we imaged carries spatial information that was not found in the imaged neurons and in their interactions.”

*Reviewers’ comment:*

** Lines 209-211 "response field position was differentially distributed in astrocytes compared to neurons, suggesting that CA1 astrocytes do not merely mirror position information encoded in CA1 pyramidal neurons." I think this is problematic because the astrocyte population is comprised of processes from the same astrocytes, whereas the neurons are independent somas. Furthermore, the population of independent astrocytes is much smaller than the population of neurons (see fig 2B versus 4C, right). Even though the authors have shown that processes from the same astrocyte can have distinct PFs, they are still more correlated than independent astrocytes. These factors will influence the population map of the corridor, making it difficult to conclude that astrocytes are differentially distributed. Again, they also did not record from all neurons or all astrocytes, and the numbers of independent astrocytes and neurons is not matched.*

Author response: the sentence indicated by the referee was deleted in response to one of the previous comments. We removed any argument about astrocytes not mirroring position information encoded in neurons based of position of the astrocytic and neuronal place fields.

*Reviewers’ comment:*

** I think the authors should expand their discussion to include what they think the downstream effects of the astrocytic spatial code is for? Obviously, the neuronal spatial code is projected out to other brain regions and used there for certain computations. But the astrocytic spatial code remains within the hippocampus. What is its ultimate function? Is the brain using this information to actually help encode external space, and if so, how? Or possibly it is serving modulatory role within the CA1 itself. If we were to inactivate the astrocytes, what would be the prediction? Would the neuronal spatial code be affected? An expansion on these ideas would be very useful for the discussion section.*

Author response: thanks for encouraging us to discuss these important points. We expanded the text at lines 321-352 to address the reviewer’s comment. The new text reads:

“Can complementary spatial information encoded in astrocytic calcium dynamics contribute to neuronal computation? If so, how? Although our data do not directly address these questions, previous work in other brain regions reported that astrocytic calcium dynamics largely mirror the activity of nearby neurons (7, 10, 11) and that astrocytic signals translate into changes in neuronal excitability and synaptic transmission through various mechanisms (reviewed in (1, 50-52)). In this scenario, changes in synaptic transmission and neuronal excitability induced by astrocytic calcium dynamics that simply mirror neuronal information would only modulate neural activity *without providing further information*, as all the activity-dependent information is already encoded in the neuronal activity. For example, if the neuronal tuning curve and the astrocytic-induced change in neural function are similarly modulated by the animal’s position, no additional dependence of neuronal function by position would be introduced by astrocyte-neuron interactions. Conversely, our findings imply that astrocytic calcium dynamics carrying *complementary information* to that of neurons enable modulations of synaptic transmission and neuronal gain which could increase the computational capability of neural circuits (53, 54). For example, changing the gain of neurons with a coordinate system complementary to that regulating its tuning function has been shown to endow neural networks with richer computations (54, 55). Moreover, targeted dynamic control of neural excitability (e.g., changing the gain of a subset of neurons in the network rather than the whole network) can greatly increase the dynamic repertoire and coding capabilities of circuits, for example by making it possible to reach different attractors from a similar set of initial conditions (56). We thus propose that the complementary place-dependence of the astrocytic calcium dynamics and the place-dependence of astrocytic-neuron interactions reported here facilitate the emergence of dynamic, context-dependent changes in population coding of CA1 neurons. Within this scenario, local neuromodulation of the space-encoding properties of projecting CA1 neurons by astrocytes could affect hippocampal output. Future experiments involving selective perturbation (e.g., activation or inactivation) of astrocytic calcium signaling will be needed to test this hypothesis. Our work calls for a re-examination of the theory of place coding and of brain population codes in light of the opportunities offered by complementary astrocytic information coding. We propose that the complementary regulation of astrocytic calcium activity and of its interaction with neurons may reflect a general principle of how the brain encodes information. This conclusion may extend beyond the hippocampus and spatial navigation to other brain regions and cognitive tasks and it will need to be included in the conceptualization of brain function.”

*1 Fenton, A. A. et al. Attention-like modulation of hippocampus place cell discharge. J Neurosci 30, 4613-4625, doi:10.1523/JNEUROSCI.5576-09.2010 (2010).*

*2 Sheffield, M. E. & Dombeck, D. A. Calcium transient prevalence across the dendritic arbour predicts place field properties. Nature 517, 200-204, doi:10.1038/nature13871 (2015).*

*3 Doron, A. et al. Hippocampal Astrocytes Encode Reward Location. bioRxiv, 2021.2007.2007.451434, doi:10.1101/2021.07.07.451434 (2021).*

*4 Calin-Jageman, R. J. & Cumming, G. Estimation for Better Inference in Neuroscience. eNeuro 6, doi:10.1523/ENEURO.0205-19.2019 (2019).*

***Reviewer #3***

*Reviewers’ comment:*

*Curreli et al. investigated how hippocampal astrocyte calcium signals are involved in spatial information encoding. The authors expressed a genetic calcium indicator in the hippocampal astrocytes of adult mice and found that astrocyte calcium activities from somata and processes both respond to spatial cues in a virtual reality environment with two-photon in vivo imaging. The authors further examined the neuronal activities in the same virtual reality setting and showed that calcium signals of astrocytes and neurons appear correlated and convey synergistic information.*

*Increasing evidence has demonstrated the importance of astrocyte calcium signaling in regulating neural circuits and animal behavior. Yet, whether astrocytes utilize their diverse calcium signals to encode spatial information is still unclear. Thus, the work by Curreli et al. is novel from this perspective. However, some of the conclusions drawn from the data are open to interpretation and there are several major issues that the authors need to adequately address. The authors also did not rule out the possibility that astrocytes solely imitate or react to changes of neighboring neurons and therefore seem to be responsive to spatial cues. Without further supporting evidence, the discovery of "astrocytic place cells" is unconvincing.*

Author response: we thank the referee for their interest in our manuscript. We performed an extensive set of new experiments and analyses to address all of their points (see responses to specific comments below).

*Major points:*

*Reviewers’ comment:*

*1. The authors imaged astrocyte calcium signals in a virtual reality with a monodirectional or bidirectional corridor, which significantly limits the exploration dimension and spatial information that are normally contained in a real environment. Therefore, instead of being responsive to the spatial information as suggested by the authors, detected astrocyte calcium signals could be induced simply by the visual stimuli such as images, lighting, shapes etc from the screen of the virtual reality setting. Without further evidence, it is unconvincing to conclude that there are "astrocytic place cells" and their calcium signals encode spatial information.*

Author response: we appreciate the Referee’s comment. We have conducted a series of new analyses to address this important point (new Fig. 4, new extended data Fig. 10, new extended data Fig. 17, and new extended data Fig. 18). Specifically, our virtual corridor was characterized by the alternation of three different patterns (grid, vertical lines, and circles) similarly to corresponding original experiments with neurons, see Harvey et al. Nature 2009, Dombeck et al. Nat. Neurosci. 2010, and Sheffield et al. Nature 2015. The three patterns covered the whole length of the virtual corridor (180 cm) and each pattern was presented for 60 cm of the corridor. Within each of these 60 cm-long visual cues, the visual stimuli associated with each pattern were periodically repeated (Fig. 1C). We reasoned that if astrocytes responses in the virtual reality corridor were only modulated by visual cues regardless of the position in which the visual stimulus was provided, then astrocytes calcium responses should not have the power to discriminate between spatial locations within the 60 cm-long spatial interval in which a single visual cue was presented. In such case, the astrocytic responses would not carry spatial information above and beyond the one that is inherited from the information they carry about the identity of the visual cue. To test whether astrocytic signals carried spatial information that cannot be possibly attributed to visual cue modulation, we randomly shuffled the relationship between position and astrocytic signals within each visual cue. This random data shuffling preserves cue information carried by the astrocytes, but destroys all the genuine spatial information they carry above and beyond visual cue information. The difference between the information carried by the real, unshuffled, responses and the information carried by the shuffled responses quantifies the amount of spatial position information carried by the astrocytes that cannot be possibly attributed to spatial cue tuning. We found that a large fraction (~ 50 to 70 %, new Fig. 4B-C) of the total spatial information carried by the astrocytic ROIs could not be possibly explained by visual cue modulation. Moreover, when analyzing individual ROIs, we found that a large fraction (~ 50 to 60 %, new Fig. 4A) of the ROIs that carried significant spatial information carried a significant amount of spatial information that could not be explained by visual cue tuning. The random shuffling analysis was performed by dividing each visual cue in N spatial intervals (N was systematically varied from 2 to 6) and the results were qualitatively similar over this range of parameter values (new Fig. 4). Importantly, we confirmed the results of these analysis on our dataset of neuronal and astrocytic simultaneous recordings (new extended data Fig. 17, new extended data Fig. 18).

Thus, these analyses demonstrated that the majority of the amount of spatial information carried by an astrocytic simultaneously recorded population is genuine spatial information that cannot be attributed to visual cue tuning, and the large majority of astrocytic ROIs carry genuine spatial information that cannot be explained by their tuning to visual cues.

These results are reported in Figure (new Fig. 4, new extended data Fig. 10, new extended data Fig. 17, and new extended data Fig. 18) and described in the text at lines 157-182 and 215-222. The new text reads:

Lines 157-182

“Our virtual corridor was characterized by the alternation of three different patterns (grid, vertical lines, and circles) similarly to (16, 17, 24). The three patterns covered the whole length of the virtual corridor (180 cm) and each pattern was presented for 60 cm of the corridor. Within each of these 60 cm-long visual cues, the visual stimuli associated with each pattern were periodically repeated (Fig. 1C). Can the different visual cues account for the modulation of spatial information that we observed in astrocytes? We reasoned that if astrocytes responses in the virtual reality corridor were only modulated by visual cues regardless of the position in which the visual stimulus was provided, then astrocytes calcium responses should not have the power to discriminate between spatial locations within the 60 cm-long spatial interval in which a single visual cue was presented. In such case, the astrocytic responses would not carry spatial information above and beyond the one that is inherited from the information they carry about the identity of the visual cue. To test whether astrocytic signals carried spatial information that cannot be possibly attributed to visual cue modulation, we randomly shuffled the relationship between position and astrocytic signals within each visual cue. This data shuffling procedure preserves cue information carried by the astrocytes but destroys all the genuine spatial information they carry above and beyond visual cue information. The difference between the information carried by the real, unshuffled, responses and the information carried by the shuffled responses quantifies the amount of spatial position information carried by the astrocytes that cannot be possibly attributed to spatial cue tuning. Analyzing individual astrocytic ROIs (Fig 4A), we found that a large fraction (~ 50 to 60 %) of spatially modulated ROIs carried significantly more information than what could be solely explained by visual cue identity. Moreover, when decoding the animals’ position from astrocytic population vectors (Fig 4B-C, Extended data Fig. 10), we found that the majority (~ 55 to 65 %) of the decoded information was genuinely information about position. We performed both analysis dividing each visual cue in a number of spatial bins that was systematically varied from 3 to 6, leading to an overall spatial granularity varying from 9 to 18, and obtaining qualitatively similar results across granularities.”

Lines 215-220

“Importantly, we also found that a large fraction of astrocytic and neuronal ROIs showing spatial modulation carried a significant amount of spatial information that could not be explained by visual cue tuning (Extended data Fig. 17A, B). Moreover, when analyzing population vectors using a support vector machine decoder, the majority (~ 60 to 80 %, Extended data Fig. 18) of the total spatial information carried by either astrocytic or neuronal ROIs could not be possibly explained by visual cue modulation.“

*Reviewers’ comment:*

*2. Astrocytes display spontaneous calcium signals that occur in the absence of external stimuli. To rule out the possibility that observed astrocyte calcium responses in the virtual reality are not due to random activities, additional controls are warranted. For example, what are the baseline calcium activities of the previously identified "place cells" without virtual reality? How do "astrocytic place cells" respond to the same virtual reality pattern, e.g. record with only displaying the vertical lines? How do they react to alternating virtual reality patterns, e.g. record with mesh pattern -> vertical lines -> mesh pattern? These experiments will help to clarify whether the calcium responses of astrocytes are specific to certain spatial cues, which is expected for a place cell.*

Author response: we thank the Referee for raising these points.

Regarding the quantification of spontaneous calcium activities of astrocytes: in the new extended data Fig. 4B and new extended data Fig. 13B, we now show the amplitude of astrocytic calcium activities in the absence vs. presence of virtual reality. We found that astrocytic calcium signals were smaller in the absence vs. presence of virtual reality, indicating that the events that we recorded during virtual navigation were fundamentally different from those occurring spontaneously (see also response to point #3 of referee #1). We modified the text at lines 89-91 and 194-196 to describe the findings presented in the new figures.

Regarding how astrocytes place cells respond to the same visual stimulus when presented at different spatial location along the virtual corridor: we performed an extensive analytical investigation to clarify this important point (see response to point #1 of this referee). We found that most of the spatial information carried by the astrocytic ROIs cannot be accounted for by their tuning to visual cues (see response to point #1 of this referee). These results (new Fig. 4, new extended data Fig. 10, new extended data Fig. 17, and new extended data Fig. 18) demonstrate that the majority of calcium response of astrocytes bring genuine spatial information. The description of these findings is now provided in the text at lines 157-182 and 215-222.

*Reviewers’ comment:*

*3. In Fig. 4, both neurons and astrocytes were imaged "in the CA1 pyramidal layer" and their calcium signals were analyzed to reveal the spatial correlation. However, there are very few astrocytes present in the pyramidal layer, so it is unclear how both cell types were imaged simultaneously with two-photon microscopy. If neurons and astrocytes were indeed captured from the same focal plane, then the authors need to provide representative images. Fig. 4A only shows astrocytes but not neurons. If, however, neurons and astrocytes were sampled from different focal planes, then the authors have to indicate so and take the depth difference into consideration for subsequent spatial analysis in Fig. 4F. The current presentation and description are misleading.*

Author response: astrocytes and neurons were imaged in the same focal plane in the CA1 pyramidal layer. As requested by the Referee, we now provide representative images (new Fig 5A-B) of imaged astrocytes and neurons. We also modified the Methods at lines 551-553 and 579-580 to clarify the recording conditions, which we used.

*Minor points:*

*Reviewers’ comment:*

*1. The cortex above the hippocampus was aspirated to facilitate in vivo imaging. However, astrocytes may become reactive and change calcium signals due to the invasive surgical procedure, this possibility should be tested with astrocyte reactive marker staining or morphological analysis.*

Author response: we performed the experiment requested by the Referee. Specifically, we stained for the prototypical marker of astrocyte reactivity, GFAP, sections of fixed tissue from animals implanted with the chronic hippocampal window (new extended data Fig. 1). As controls, we used the contralateral non-implanted hemisphere from the same animals. We quantified GFAP signals in implanted and control hemispheres in three regions: the Stratum Oriens, the Stratum Pyramidale, and the Stratum Radiatum. We found similar GFAP immunoreactivity in the Stratum Pyramidale and Radiatum in implanted hemispheres compared to controls (new extended data Fig. 1E, F). In contrast, we observed increased GFAP immunoreactivity in the Stratum Oriens in implanted hemispheres compared to controls (new extended data Fig. 1E, F). These results are in line with those reported in previous publications (Gu et al. 2014, Attardo et al. 2015), which reported no astrocyte reactivity in the Stratum Pyramidale, where imaging was performed, and some astrocyte reactivity in a small region close to the glass coverslip of the implant (in the Stratum Oriens). We added a figure (new extended data Fig. 1E, F) and modified the text in the Results section (lines 52-63) and in the Methods (lines 940-953) to describe these findings.

*Reviewers’ comment:*

*2. As the authors already pointed out, the properties of astrocyte calcium signals from different cellular compartments (e.g. somata vs. branches vs. processes) are fundamentally different. It is interesting that the distribution of field position of ROIs from soma are similar to the ones from the processes. However, it is unclear how many ROIs were identified from the processes belonging to the same astrocytes. It is not surprising that ROIs from the same cellular compartment of the same astrocytes are highly correlated, as shown in Fig. 2F and Fig. 2G. Thus, the authors should separate intracellular correlation from intercellular correlation. Combing all ROIs together may overlook the diversity of astrocyte calcium signals. In addition to the number of ROIs and mice, the authors should also clarify the number of cells in the analysis.*

Author response: following the Reviewer’s comment, we report the analysis (originally shown in Fig. 2F and Fig. 2G) for pairs of ROIs belonging to the same astrocyte (A_same_, Fig. R3) and for pairs of ROIs belonging to different astrocytes (A_other_, Fig. R3). We found that the absolute distance of the response field center (Fig. R3B) increased with distance and the Pearson correlation (Fig. R3D) decreased with distance for pairs of ROIs belonging to the same astrocyte, similarly to what we reported in the original Fig. 2G and Fig. 2F for all pairs of ROI and pairwise distances < 35 µm (Fig. R3A, C). ROI pairs belonging to different astrocytes displayed results similar to those originally presented in Fig. 2G and Fig. 2F for all pairs of ROI and pairwise distances > 35 µm. Altogether, these results are in line with the observation that hippocampal astrocytes have non-overlapping territories with a characteristic diameter of ~ 50-60 µm (Bushong et al. 2002 & Halassa et al. 2007).

The number of cells included in the analysis is now reported in the main text (lines 396).

**Figure R3. Astrocytic event probability during virtual navigation.** (A-B) Difference between response field position for pairs of astrocytic ROIs containing reliable spatial information across the whole FOV as a function of pairwise ROI distance. In (B) ROI pairs were grouped according to belonging either to the same astrocyte (A-A_same_, red) or to different astrocytes (A-A_other_, blue). In (C-D) same as in (A-B) for Pearson correlation values. In all panels solid lines and the shaded regions indicate mean ± s.e.m, respectively. Data from 41 cells in which there was significant spatial modulation in at least one ROI, over 7 imaging sessions in 3 animals.

*Reviewers’ comment:*

*3. In Fig 3B, because the "chance level" is extremely low so it doesn't appear in the bar graph. The y axis should be adjusted to show all the data, e.g. starting from -0.1.*

Author response: thanks for pointing our attention to this figure panel. We adjusted the y axis to now show all the data (new Fig. 3B).

References

Attardo A, Fitzgerald JE, Schnitzer MJ. Impermanence of dendritic spines in live adult CA1 hippocampus. Nature. 2015;523(7562):592-6.

Bushong EA, Martone ME, Jones YZ, Ellisman MH. Protoplasmic astrocytes in CA1 stratum radiatum occupy separate anatomical domains. J Neurosci. 2002 Jan 1;22(1):183-92.

Gu L, Kleiber S, Schmid L, Nebeling F, Chamoun M, Steffen J, et al. Long-term in vivo imaging of dendritic spines in the hippocampus reveals structural plasticity. J Neurosci. 2014;34(42):13948-53.

Halassa MM, Fellin T, Takano H, Dong JH, Haydon PG. Synaptic islands defined by the territory of a single astrocyte. J Neurosci. 2007 Jun 13;27(24):6473-7.

Harvey CD, Collman F, Dombeck DA, Tank DW. Intracellular dynamics of hippocampal place cells during virtual navigation. Nature. 2009;461(7266):941-6.

Dombeck DA, Harvey CD, Tian L, Looger LL, Tank DW. Functional imaging of hippocampal place cells at cellular resolution during virtual navigation. Nat Neurosci. 2010;13(11):1433-40

Sheffield ME, Dombeck DA. Calcium transient prevalence across the dendritic arbour predicts place field properties. Nature. 2015;517(7533):200-4.
