# Supplementary material for: Complementary encoding of spatial information in hippocampal astrocytes
Source: PLoS Biol. 2022 Mar 3;20(3):e3001530. doi: 10.1371/journal.pbio.3001530 (PMC8893713; doi:10.1371/journal.pbio.3001530)
Supplement: Attachment — Submitted filename: Point-by-point response.docx [file pbio.3001530.s039.docx]

**Point-by-point response to the referee’s comments**

**Reviewer #3**

*Referee’s comment: Curreli et al. has included additional analyses in the revised manuscript to address most of my questions. One issue that I encourage the authors to seriously consider, which was also raised by other reviewers is to tune down their claims that astrocytes and neurons encode or process different spatial information. Since astrocytes are fundamentally different from neurons, the fact that astrocyte calcium signaling exhibit distinct properties from neurons could likely be due to intrinsic molecular machinery operated in the cells. That is, astrocyte and neuronal calcium signaling can encode the same information, but display in a distinct manner. In fact, transcriptomic and proteomic studies have all supported this notion. For instance, the authors concluded based on Fig. 6 that "This result proved that the population of astrocytic ROIs carries information not found in neurons or their interactions." In order to experimentally "prove" this statement, the authors should manipulate calcium signaling in either astrocytes or neurons, and analyze the consequences of animal's navigational abilities, which I believe is beyond the scope of this study. Thus, the authors should rephrase their statements to avoid misleading the readers.*

Author response: we thank the Reviewer for these suggestions. The Reviewer asks us to insert two caveats in the work.

The first regards the possibility that “astrocyte and neuronal calcium signaling can encode the same information, but display in a distinct manner” because of the major differences in their molecular and biophysical properties. While we agree that this is an interesting point to discuss in the paper (lines 284-310) and to investigate in future work, we also note that we designed the information theoretic part of our analyses specifically to be unaffected by such a possible confounder. In fact, the theorems of information theory state that if cell A and cell B exactly encode the same information but cell B displays it in a different format or manner, then the joint information jointly carried by the activity of A and B cannot be larger than the information in A or B alone, and that the amount of complementary information between A and B would be zero (Panzeri S et al. 2015). Thus, the finding that the spatial information in astrocytic-neural pairs is larger than the spatial information carried in one of the two cells (i.e., astrocytes and neurons carry complementary spatial information) speaks against the possibility that astrocytes carry the same spatial information of neurons but in a different format. Also, the finding that part of the complementary information is due to the information created by the spatial modulation of the correlations between astrocytes and neurons shows that part of the spatial information carried by the neuro-glia network is not contained in neurons or astrocytes alone, but is contained in their interactions, which again speaks against the confounder indicated by the referee. Finally, the fact that in some studies astrocytes and neurons carry largely the same information and broadly mirror each other (e.g., Schummers et al. Science 2008) speaks against the possibility that the major differences in the biophysical properties of neurons and astrocytes enforce neural and astrocytic representation of spatial information to be in different formats.

With this being said, we carefully reviewed the manuscript, modified the sentence indicated by the referee (lines 259-260), and edited the paragraph in the Discussion that raises the possibility, to be investigated in future work, that astrocytes encode the same space information as neurons but they display it in a different format (lines 285-317). Specifically, we replaced the word “prove” with “supports the hypothesis” (line 259) to avoid that a reader might misunderstand that we are referring to an experimental “proof” of the behavioral role of spatial astrocytic information.

As Reviewer #3 also noted, in the previous round of revisions we already made substantial revisions to the manuscript to address this issue under the advice of Referee #2, and we were happy to note that Reviewer #2 was satisfied by our work.

The second caveat raised by the Referee regards the discussion that proving the consequences of our findings for circuit function and animal’s navigational abilities will need manipulating calcium signaling in either astrocytes or neurons. To address this point of the Referee, we expanded the Discussion section (lines 310-316). We now discuss that showing encoding of information in cells is not enough to demonstrate that the information they encode is consequential to behavior or circuit computations, and that to causally prove the contribution of this information it is necessary to specifically manipulate either astrocytes and neurons while monitoring downstream effects on circuit functions and/or behavior.

We also note that, in the neural coding literature, using the term “encode” is a well-established nomenclature to indicate that there is a robust statistical relationship between the activity of the cell and the sensory or position variable of interest, from which information can be extracted. It is also well understood and recognized that the encoding of information does not necessarily imply that this information is used for function or behavior, and that manipulations, rather than statistical analyses, must be used to establish causality (Panzeri et al. Neuron 2017). To make sure that this concept is clear to all readers, we made small additions to the abstract and to the introduction to define that we use “encoding” to refer to statistical, rather than causal, relationships. Also, the aforementioned paragraph in Discussion clarifies that encoding is not enough to prove consequentiality of the encoded information for circuit function and behavior.

We carefully checked the whole manuscript for places in which we might have used loose language. We checked that the term “encoding” was consistently used across the manuscript. We made few minor corrections in places were the writing could have allowed a causal interpretation of our encoding findings (e.g., replaced “carries” with “encodes” at line 259 and remove “actively” before “encodes” at line 47).
